# Practical considerations in the management of patients treated with bosutinib for chronic myeloid leukemia

**DOI:** 10.1007/s00277-024-05851-4

**Published:** 2024-07-18

**Authors:** Jeffrey H. Lipton, Tim H. Brümmendorf, Kendra Sweet, Jane F. Apperley, Jorge E. Cortes

**Affiliations:** 1 Princess Margaret Cancer Centre, Toronto, Canada; 2 Department of Hematology, Oncology, Hemostaseology and Stem Cell Transplantation, Faculty of Medicine, RWTH Aachen University Hospital, Aachen, Germany; 3 Center for Integrated Oncology, Aachen Bonn Cologne Düsseldorf (CIO ABCD), Aachen, Germany; 4 Moffitt Cancer Center, Tampa, FL USA; 5 Centre for Haematology, Imperial College London, London, UK; 6 Division of Hematology and SCT, Georgia Cancer Center, Augusta, GA USA

**Keywords:** Chronic myeloid leukemia, Bosutinib, Adverse event management, Dosing strategies

## Abstract

Bosutinib is a second-generation tyrosine kinase inhibitor indicated for the treatment of patients with newly diagnosed Philadelphia chromosome–positive chronic phase chronic myeloid leukemia (CML), and for patients with Ph + chronic phase, accelerated phase, or blast phase CML resistant or intolerant to prior therapy. As is the case for all TKIs approved for treatment of CML, bosutinib is associated with adverse events (AEs) that require appropriate management to ensure adherence to treatment and optimized outcomes. The aim of this review is to provide physicians with updated practical information for the prevention and management of AEs occurring during treatment with bosutinib, including dosing strategies, based on the latest published evidence and clinical experience. Clinical studies and real-world evidence have shown bosutinib has a generally favorable safety profile, which has remained consistent across lines of therapy and in long-term reports. Adjusting the starting dose and/or modifying the dose during treatment with bosutinib are important strategies to manage AEs and improve tolerability, which are recognized within the label and in treatment guidelines. Dosing adjustment strategies to manage AEs are a recognized management approach for other TKIs in the treatment of CML and are not exclusive to bosutinib. In summary, long-term results from clinical trials and emerging real-world evidence demonstrate bosutinib has a safety profile that can largely be managed with treatment modifications and/or supportive care. Increased experience in managing toxicities and by using a personalized dosing approach may further improve adherence and outcomes with bosutinib.

**Supplementary Information:**

The online version contains supplementary material available at 10.1007/s00277-024-05851-4.

## Introduction

Bosutinib is a second-generation tyrosine kinase inhibitor (TKI) indicated for the treatment of patients with newly diagnosed Philadelphia chromosome–positive (Ph +) chronic phase (CP) chronic myeloid leukemia (CML), and for patients with Ph + CP, accelerated phase, or blast phase CML resistant or intolerant to prior therapy [1, 2]. A distinct feature of bosutinib is its lack of inhibitory activity in preclinical studies against c-kit and PDGF-R [3]. This may mean that bosutinib has a reduced rate of adverse events (AEs) such as hypopigmentation and edema, which have been associated with inhibition of c-kit and PDGF-R, respectively, with imatinib [4, 5]. This may also make bosutinib an attractive option in pediatric patients in which both c-kit and PDGF-R inhibition has been associated with growth inhibition and short stature [6]. The approved bosutinib starting dose is 400 mg once daily in the first-line setting and 500 mg once daily in the second- or later-line setting [1, 2].

Like all TKIs approved for the treatment of CML, bosutinib treatment is associated with AEs that require careful consideration and appropriate management to ensure adherence to treatment and optimized outcomes [7]. Recommendations on the management of bosutinib-associated AEs were published by an expert panel of hematologists in 2018 [8]. Since then, further information from long-term follow-up of clinical trials and real-world studies has been published [7].

The aim of this review is to provide physicians with an updated expert consensus regarding practical information for the prevention and management of AEs occurring during treatment with bosutinib, including dosing strategies, based on the latest available evidence and clinical approaches the authors have found useful in their practice.

## Specific AEs during bosutinib treatment

Bosutinib has a generally favorable safety profile and is a good option for patients with certain comorbidities [9, 10]. The distinct bosutinib safety profile is characterized by gastrointestinal (GI), hematological, hepatic, and skin toxicities and has remained consistent across lines of therapy and in long-term studies (Supplemental Table 1 and Supplemental Table 2, Online Resource 1) [11–20]. Hematological AEs such as thrombocytopenia and anemia; non-hematological AEs such as diarrhea, nausea, vomiting, abdominal pain, pyrexia, headache, and rash; and laboratory AEs, including increased alanine aminotransferase (ALT) and aspartate aminotransferase (AST) [2, 11–20], are the most commonly reported clinical AEs for bosutinib across clinical trials. The most frequent grade 3 or 4 hematological AEs include thrombocytopenia and neutropenia. Diarrhea, rash, anemia, and increased ALT, lipase, and AST represent the most common grade 3 or 4 non-hematological AEs [2, 11–20]. Most AEs occur within the first year of treatment and are transient [1, 2, 15, 20–22]. In later years, cardiac, vascular, effusion, and renal AEs may occur [21, 22]. The incidence of AEs, discontinuations due to AEs, and median dose intensity are detailed in Online Resource 1, Supplemental Table 1 for clinical trials and Supplemental Table 2 for real-world studies.

Bosutinib does not carry a black box warning, and the European LeukemiaNet advises that no strong contraindications have been identified [9]. However, bosutinib should be used with caution in certain circumstances discussed further below with respect to individual AEs. Co-administration of bosutinib with strong cytochrome P450 (CYP)3A inhibitors or inducers, which may affect plasma concentrations of bosutinib and most other TKIs, should be avoided [1, 2]. Patient education, preventative strategies, and early and prompt recognition of AEs are important for optimal management (Table 1) [23]. These strategies are discussed in more detail in the following sections.

**Table 1:** Management of adverse events

Adverse events	Education, assessment, and management
Thrombocytopenia and neutropenia	Identify and manage hematological AEs promptly
	**Before bosutinib treatment initiation** • Educate patients to recognize and report potential signs and symptoms of hematological AEs, such as bruising, fever, signs of infection, unexpected bleeding, or blood in urine or stool
	**During bosutinib treatment** • Monitor complete blood counts regularly; weekly for first few weeks then frequency of monitoring can be reduced • Assess for signs or symptoms of hematological AEs, such as bruising, fever, signs of infection, and unexpected bleeding or blood in their urine or stool • Patients on stable, long-term treatment may only need to be monitored every 2–3 months
	**Management** • Consider bosutinib dose reductions or interruptions for grade 3/4 or persistent neutropenia and thrombocytopenia • Growth factors and other supportive care can also be used [1, 2, 7, 23, 24] • If ANC < 1000 × 10^6^/L or platelets < 50,000 × 10^6^/L, interrupt bosutinib until ANC ≥ 1000 × 10^6^/L and platelets ≥ 50,000 × 10^6^/L [1, 2] • Resume treatment with bosutinib at the same dose if recovery occurs within 2 weeks [1, 2] • If blood counts remain low for > 2 weeks, upon recovery, reduce dose by 100 mg and resume treatment [1, 2] • If cytopenia recurs, reduce dose by an additional 100 mg upon recovery and resume treatment [1, 2]
CV events	Exercise caution in patients with cardiac disorders
	**Before bosutinib treatment initiation** • Assess and manage underlying cardiac and vascular risk factors (eg, prophylactic treatment in line with guidelines for that condition, lifestyle modifications, and correction of serum electrolytes) [25] • Involve a cardiologist, cardio-oncologist, or other relevant specialist (eg, endocrinologist for diabetes) for high-risk patients or when efforts to manage comorbidities are insufficient • Educate patients to recognize and promptly report signs and symptoms of cardiac or vascular AEs during treatment or, if severe, attend the emergency room [7, 26]
	**During bosutinib treatment** • Assess serum electrolytes at baseline and monitor throughout • Administer bosutinib with caution in patients with history/predisposition for QTc prolongation, and a baseline electrocardiogram is recommended [1, 2, 8] • Avoid concomitant administration of bosutinib with QTc-prolonging drugs, eg, domperidone, chloroquine, clarithromycin, methadone, and anti-arrhythmic medicines [2, 26]
	**Management** • Consider patient risk factors, concomitant medications, and lifestyle factors • Cardiac or vascular AEs can mostly be managed with concomitant medications, dose interruptions, and dose reductions, as well as regular monitoring throughout bosutinib treatment [7, 21, 23]
Renal dysfunction	**Before bosutinib treatment initiation** • Assess risk factors for renal dysfunction • SLC22A2 808G > T polymorphism of the OCT2 transformer has been associated with increased creatinine levels with the use bosutinib • Assess renal function at baseline. For moderate impairment (creatinine clearance 30–50 mL/min), start bosutinib at 300 mg QD for 1L patients and 400 mg QD for 2L patients. For severe impairment (creatinine clearance < 30 mL/min), start bosutinib at 200 mg QD for 1L patients and 300 mg QD for 2L patients [1, 2] • Educate patients to recognize and promptly report any changes in urinary output or frequency [7]
	**During bosutinib treatment** • Renal events tend to occur late in treatment course (> 1 year) [22, 27] • Monitor renal function during treatment, particularly in patients with pre-existing renal impairment or risk factors for renal dysfunction • Remind patients to recognize and promptly report any changes in urinary output or frequency
	**Management** • Consider whether patient risk factors and concomitant medications are possible underlying causes • Manage with concomitant medications, dose interruptions, dose reductions, and regular monitoring [7, 23]
Skin toxicity	**During bosutinib treatment** • Educate patients about behavioral changes, such as avoiding long, hot baths and keeping adequately hydrated, which will reduce skin AEs [8, 26]
	**Management** • Manage severe cases of rash with topical or systemic steroids or antibiotics • Consider bosutinib dose reductions, dose interruptions; permanent discontinuation should be considered when management is ineffective or in instances of recurrent, severe skin toxicity [1, 2, 24] • Consult a dermatologist, if appropriate
Diarrhea	**Before bosutinib treatment initiation** • Educate patients that diarrhea can start early in treatment (from day 2) but should improve over time [18] • Advise about appropriate preventive and mitigating measures, such as taking bosutinib with food and water, keeping hydrated, and avoiding foods that may exacerbate symptoms • Consider starting bosutinib at a lower dose followed by a dose escalation strategy, as tolerated, if appropriate
	**During bosutinib treatment** • Maintain close communication and regular follow-up
	**Management** • Diarrhea during bosutinib treatment is manageable with supportive care and/or dose modifications [1, 2, 26] • Pharmacologic measures for diarrhea treatment include use of anti-diarrheal medications and/or fluid replacement [1, 2, 8, 26] • Avoid PPIs due to a drug–drug interaction that may reduce efficacy [1, 2] • Short-acting antacids or H2 blockers can be administered but should be separated by more than 2 h with administration of bosutinib [1, 2] • Consider a dose reduction followed by a dose escalation strategy, as tolerated • For grade 3–4 diarrhea, bosutinib should be interrupted and may be resumed upon recovery to grade ≤ 1 [1, 2]
Other GI toxicities • Nausea • Vomiting • Abdominal pain	**Before bosutinib treatment initiation** • Educate about appropriate preventive and mitigating measures, such as taking bosutinib with food and water, keeping hydrated, and avoiding certain foods that may exacerbate symptoms
	**Management** • Monitor and follow up regularly and intervene promptly [1, 2, 8, 26] • Anti-emetic drugs and/or fluid replacements can be used to manage symptoms • Dose adjustments for bosutinib can be offered although are not frequently needed
Elevated liver enzymes • ALT increased • AST increased	Transaminase elevations usually occur early in treatment (~ 1 month)
	**Before bosutinib treatment initiation** • Assess risk factors for hepatic dysfunction • Assess hepatic function at baseline. Starting dose for patients with mild (Child–Pugh A), moderate (Child–Pugh B), or severe (Child–Pugh C) impairment is 200 mg QD (1L and 2L) [1] • Educate patients to recognize and promptly report potential signs and symptoms, such as dark urine, pale stools, or jaundice • Advise patients to avoid excess alcohol consumption and hepatotoxic agents
	**During bosutinib treatment** • Monitor liver enzymes regularly (at least monthly in the first 3 months)
	**Management** • Patients with pre-existing liver dysfunction should start treatment at a lower dose (usually 200 mg QD) and adjust as needed depending on response/tolerability • Consider other potential causes, such as infection, other medications, or alcohol overuse, as part of investigation of elevated ALT and AST • If elevations in liver transaminases > 5xULN occur, interrupt bosutinib until recovery to ≤ 2.5xULN and resume at 400 mg QD thereafter • If recovery takes > 4 weeks, discontinue bosutinib • If transaminase elevations ≥ 3xULN occur concurrently with bilirubin elevations greater than 2xULN and ALP < 2xULN (Hy’s law case definition), discontinue bosutinib • No specific supportive therapies are currently recommended, but alcohol consumption and co-administration of other hepatotoxic agents should be avoided [7, 8]
Pulmonary toxicities	**Before bosutinib treatment initiation** • Screen for pleural effusion risk factors (age ≥ 65 years, history of effusion events, history of hypertension, history of tobacco use, history of pulmonary events, and treatment with bosutinib) [20, 21]
	**During bosutinib treatment** • Regular screening for pleural effusions is not required; however, patients should be monitored for symptoms of shortness of breath, particularly on exertion or orthopnea, or edema [1, 2, 21, 23]
	**Management** • Manage using supportive care, including diuresis or steroids, and dose modifications [1, 2, 23]

Modified from Gambacorti-Passerini et al. 2020 [26]

*1L* first line; *2L* second line; *AE* adverse event; *ALP* alkaline phosphatase; *ALT* alanine aminotransferase; *ANC* absolute neutrophil count; *AST* aspartate aminotransferase; *QD* once daily; *QTc* corrected QT interval; *TKI* tyrosine kinase inhibitor; *ULN* upper limit of normal

### Hematological AEs

The most common hematologic AEs are thrombocytopenia, neutropenia, and anemia, but other hematologic AEs are frequently reported such as leukopenia [7, 15, 20] (Supplemental Table 1 and Supplemental Table 2, Online Resource 1). Most of these hematological AEs occur early in bosutinib treatment (within the first year, most commonly in the first few months), and most do not lead to discontinuations [16].

Of these, thrombocytopenia is the most common: 28% (any grade) in BELA, 36% in the 5-year analysis of BFORE, 42% in the > 10 years of follow-up of a phase 1/2 study, and 11% in BYOND [15, 16, 18, 20]. Corresponding figures for grade 3–4 are 13%, 14%, 25%, and 8%, respectively.

Myelosuppression is perhaps the most common cause of treatment discontinuation/interruption or dose reduction. Regular monitoring with complete blood counts is recommended (Table 1). These are typically performed more frequently during the first few weeks (eg, every 1–2 weeks) until counts are stable. The frequency of monitoring can then be decreased. Furthermore, patients and clinicians should be aware of potential signs or symptoms of hematological AEs, such as bruising, fever, signs of infection, and unexpected bleeding or blood in the urine or stool. If hematological AEs were a feature of previous TKI treatment, bosutinib should be started at a lower dose (100 or 200 mg daily) and increased according to tolerability and efficacy. Bosutinib dose reductions or interruptions should be considered for severe (grade ≥ 3) or persistent neutropenia and thrombocytopenia, and growth factors and other supportive care can also be used [1, 2, 7, 23, 24]. The combination of dose adjustments with supportive care may allow bosutinib treatment to be continued. After a few weeks, blood counts may spontaneously return to normal/baseline levels or once a cytogenetic response is achieved [23]. Patients on stable, long-term treatment may only need to be monitored every 2–3 months. Finally, in some patients, dose re-escalation may be possible if needed after the first several months, depending on the response to therapy.

#### Cardiovascular events

Although cardiac and vascular AEs occur in patients on bosutinib, most patients are able to continue treatment (Supplemental Table 1 and Supplemental Table 2, Online Resource 1). In two long-term follow up reports of clinical trials, ≤ 11% of patients experienced cardiac events and ≤ 9% had vascular events. This led to treatment discontinuation in ≤ 0.9% and ≤ 1.2% of patients, respectively. In these studies, the exposure-adjusted incidence rates were ≤ 0.044 for cardiac events and ≤ 0.035 for vascular events [20, 21]. Bosutinib has been compared with imatinib in two randomized phase 3 studies (BELA and BFORE), and rates of cardiovascular and vascular AEs were found to be similar between the two treatments in each trial. In BELA, after 24 months follow-up, cardiovascular AEs were reported by 10% of bosutinib-treated patients and 8% of imatinib-treated patients, with no statistically significant differences observed between the groups [28]. After 5 years follow-up in BFORE, rates of exposure-adjusted treatment-emergent cardiac AEs were 0.031 (95% CI 0.020–0.046) with bosutinib and 0.029 (95% CI 0.018–0.043) with imatinib; rates of exposure-adjusted treatment-emergent vascular AEs were 0.023 (95% CI 0.014–0.036) with bosutinib and 0.011 (95% CI 0.005–0.021) with imatinib; and rates of exposure-adjusted treatment-emergent hypertension AEs were 0.034 (95% CI 0.022–0.048) with bosutinib and 0.037 (95% CI 0.025–0.053) with imatinib [29]. Given the low rates of cardiovascular events, bosutinib is the preferred option in patients with cardiac or vascular comorbidities [29, 30].

As with all TKIs, the assessment and management of underlying cardiac or vascular risk factors prior to initiating treatment with bosutinib is essential, eg, optimal management of co-morbidities, prophylactic treatment in line with guidelines for that condition, lifestyle modifications, and correction of serum electrolytes (Table 1) [25]. In high-risk patients or instances where efforts to manage comorbidities are not sufficient, involvement of a cardiologist, cardio-oncologist, or other relevant specialist (eg, endocrinologist for diabetes, tobacco cessation for smoking, etc.) is often helpful. Patients should also be educated on signs and symptoms of cardiac or vascular AEs and to promptly report these to their physician, or an emergency room if severe, at any stage during their treatment [7, 26].

Bosutinib should be administered with caution in patients who have a history of or predisposition for QTc prolongation, and a baseline electrocardiogram is recommended [1, 2, 8]. Serum electrolytes (potassium, magnesium, etc.) should be assessed at baseline and monitored throughout. Although the risk of QTc prolongation is low and not included as a black box warning, where possible, concomitant administration of bosutinib with QTc-prolonging drugs should be avoided, eg, domperidone, chloroquine, clarithromycin, methadone, and anti-arrhythmic medicines [2, 26].

In the event of a cardiac or vascular AE during bosutinib treatment, the patient’s risk factors, concomitant medications, lifestyle factors, and other factors should be considered and adequately managed. Additionally, cardiac or vascular AEs can mostly be managed through concomitant medications, dose interruptions, and dose reductions, as well as regular monitoring throughout bosutinib treatment [7, 21, 23].

#### Renal dysfunction

Renal AEs, in the form of increases from baseline in serum creatinine and decreases in estimated glomerular filtration rate (eGFR), have been reported during treatment with bosutinib but rarely lead to treatment discontinuations (Supplemental Table 1 and Supplemental Table 2, Online Resource 1) [27]. In a retrospective analysis of phase 1/2 and phase 3 clinical trials, renal events were reported in 13% of patients receiving second-line bosutinib and 9% of patients receiving first-line bosutinib, leading to discontinuations in 1% and 0% of patients, respectively [27].

During bosutinib treatment, renal function should be monitored at 3-month intervals, or more frequently if signs of renal impairment are present (Table 1) [7]. Renal events occur late in the treatment course (median time to first event has been reported as 497 days in the second-line setting and 421 days in the first-line setting), with an increase in incidence in later years [22, 27]. Long-term bosutinib treatment has been associated with a decrease in eGFR; however, the effect does not appear to be dose-dependent [27]. The decrease in eGFR appears to be reversible and similar to the renal decline observed with long-term imatinib treatment [27]. Therefore, there is controversy as to whether this reflects kidney damage versus interference with creatinine reabsorption. Imatinib increases serum creatinine by reversibly inhibiting its secretion from the glomerular tubules [31]; imatinib does this via inhibition of the active transporters organic cation transporter 2 (OCT2) and multidrug and toxin extrusion 1 (MATE1) [32]. An increase in serum creatinine was observed to occur with bosutinib via inhibition of OCT2, and the *SLC22A2* 808G > T polymorphism of OCT2 has been associated with increased creatinine levels during bosutinib use [33]. Thus, perhaps the reversible changes in eGFR with bosutinib are due to changes in tubular transport of serum creatinine and not a decrease in filtration rates. Nevertheless, in a renal impairment study, increases in bosutinib exposure were observed in patients with moderate or severe renal impairment compared with those with normal renal function [1, 2, 27]. Consequently, renal function must be monitored at baseline and during treatment, particularly in patients with pre-existing renal impairment or risk factors for renal dysfunction. Dose adjustments are recommended in patients with baseline and treatment-emergent renal impairment [1, 2, 24, 27].

In the event of a renal AE during bosutinib treatment, patient risk factors and concomitant medications should be investigated and properly managed. Additionally, renal AEs can be managed through dose interruptions and dose reductions [7, 23].

#### Skin toxicity

Bosutinib has been associated with dermatologic AEs, in particular rash (Supplemental Table 1 and Supplemental Table 2, Online Resource 1). Rash AEs have been reported in 23% of patients after 5 years of follow-up in BFORE, in 15% of patients in BYOND, and in 36% of patients in the second-line setting in a phase 1/2 trial [16, 18, 20]. Most instances of skin AEs were grade 1 or 2, typically occurring early in treatment (median time to first event was 57.5 days) and rarely (1%) leading to treatment discontinuation [15, 16].

Severe cases of rash can be managed with topical or systemic steroids; in addition, in severe cases dose reductions and treatment interruptions should be considered. Permanent discontinuation should be considered when these measures are ineffective or in instances of recurrent severe skin toxicity (Table 1) [1, 2, 24]. The management of dermatologic toxicities is greatly assisted by behavioral changes such as avoiding long, hot baths, avoiding unprotected sun exposure, and keeping adequately hydrated, and patient education is critical to helping elicit the required adjustments [8, 26].

#### Diarrhea

Diarrhea is the most common AE reported with bosutinib (Supplemental Table 1, Online Resource 1), and caution is therefore advised in patients with GI comorbidities [1, 2]. Across trials, including in long-term follow up, any-grade diarrhea occurred in ≥ 70% of patients receiving bosutinib in the first- and later-line settings [13, 15–18, 20]. However, a lower incidence of diarrhea (52%) has been reported in a real-world study of patients with previously treated CML [34] (Supplemental Table 2, Online Resource 1). Unlike imatinib, body mass index (BMI) does not appear to impact the incidence of diarrhea [35].

Although the incidence of diarrhea is high, most cases are mild in severity, are transient in nature, and rarely result in treatment discontinuation [13, 15, 18, 20] (Supplemental Table 1, Online Resource 1). Similar results have been observed in a real-world study [34] (Supplemental Table 2, Online Resource 1). Health-related quality of life is not negatively impacted by diarrhea or chronic diarrhea in patients treated with bosutinib [36].

Diarrhea typically starts in the first few days or weeks of treatment with bosutinib. In BYOND for example, the median time of diarrhea onset was 2 days with a median duration of 8 days [18]; thus, it is important patients and physicians are aware that although diarrhea occurs early in treatment, it improves over time. At the start of treatment patients should be advised to take the drug when they have access to a restroom. Diarrhea is also overwhelmingly grade 1 or 2 and responsive to antidiarrheal medications when needed, although most patients do not need to use these agents. Patient education and preventative measures are especially important early in treatment, and patients should receive regular follow up (Table 1) [23]. Diarrhea during bosutinib treatment is manageable with supportive care and/or dose modifications [1, 2, 26]. Patient education that includes dietary advice (eg, avoiding high-residue or fatty foods, considering lactose intolerance when applicable) as well as maintaining close communication and monitoring of patients are crucial for supporting continued bosutinib treatment [8, 24, 26]. Other mitigating measures for diarrhea include taking bosutinib with food and water and keeping well hydrated. Pharmacologic measures for diarrhea treatment include use of anti-diarrheal medications and/or fluid replacement [1, 2, 8, 26]. Diarrhea is frequently managed, or even prevented, by starting at a lower dose followed by a dose escalation strategy, as tolerated, or by dose modifications after initiation of treatment (Supplemental Table 1, Online Resource 1). For grade 3–4 diarrhea, bosutinib should be interrupted and may be resumed upon recovery to grade ≤ 1 [1, 2]. Concomitant use with proton pump inhibitors (PPIs) should be avoided due to a drug–drug interaction that reduces bosutinib serum concentration and may reduce efficacy [1, 2]. Short-acting antacids or H2 blockers can be administered instead of PPIs but should be separated by more than 2 h with administration of bosutinib [1, 2]. Diarrhea usually improves over time; thus, adequate management in the first few weeks is important in allowing patients to adjust to, and tolerate, long-term therapy.

#### Other GI toxicities

Other GI toxicities, including nausea, vomiting, and abdominal pain, are less frequently observed than diarrhea with bosutinib treatment and are mostly grade 1 or 2 in severity, rarely leading to treatment discontinuation (Supplemental Table 1 and Supplemental Table 2, Online Resource 1). Across trials, including in long-term follow up, any-grade nausea and vomiting occurred in ≤ 46% and ≤ 33% of patients, respectively, and were often managed with dose interruptions/reductions and successful rechallenge, and/or concomitant medications [13, 15, 17, 18, 22].

Mitigating measures are similar to those described for diarrhea (Table 1). Dose adjustments for bosutinib can be offered although are not frequently needed. Anti-emetic drugs are seldom needed but should be offered when appropriate, and fluid replacement should be used for patients with significant decrease in oral intake (or diarrhea). Intervention should be prompt, and physicians should closely monitor and follow-up closely with patients [1, 2, 8, 26].

#### Liver enzyme elevations

Abnormal liver function, characterized by elevations of AST and ALT, have been reported with bosutinib (Supplemental Table 1 and Supplemental Table 2, Online Resource 1); therefore, caution is warranted in patients with hepatic impairment or comorbidities. As with other TKIs, patients should be assessed for previous hepatitis B exposure [2, 8]. After a 5-year follow-up in BFORE, 44% of patients had experienced any grade liver AEs, including increased ALT (any grade, 33.6%; grade 3/4, 20.9%) and AST (any grade, 25.7%; grade 3/4, 10.4%) [20, 22]. The incidence of elevated ALT and AST in patients treated with bosutinib has been reported to be higher in those with high BMI (≥ 25 kg/m^2^) compared with those with low BMI (< 25 kg/m^2^) [35].

In clinical trials and real-world studies, ALT and AST elevations are the most common reasons for bosutinib discontinuations, although the overall rates are low, typically below 5% [13, 17, 18, 20] (Supplemental Table 1 and Supplemental Table 2, Online Resource 1). Most discontinuations occur in the first year of treatment [22].

In the BELA and BFORE trials, most AEs of increased ALT and AST were managed with dose interruptions, dose reductions, or concomitant medications [15, 22]. Successful treatment re-challenge was achieved in 73.8% of bosutinib-treated patients who were re-administered the study drug after dose interruptions and, in most instances, with dose adjustments [22].

Transaminase elevations usually occur early during treatment, with median time to first event of 28–29 days; therefore, frequent liver function monitoring is recommended for the first 3 months of treatment and as clinically indicated thereafter (Table 1) [1, 2, 15]. Dose modifications and/or transient treatment interruptions may be necessary, depending on the enzyme levels, to manage abnormal liver function [1, 2]. In cases of elevated ALT and AST, it is important other potential causes, such as infection, other medications, or alcohol overuse, should be considered as part of any investigation. There are currently no specific supportive therapies recommended, but alcohol consumption and co-administration of other hepatotoxic agents should be avoided [7, 8]. Furthermore, the NCCN guidelines recommend patients with pre-existing mild, moderate, and severe hepatic impairment start with a lower dose of bosutinib [24]. For patients with pre-existing liver dysfunction, it is recommended to initiate treatment at a lower dose, usually 200 mg daily, and adjust as needed depending on response and tolerability.

#### Pulmonary toxicities

Bosutinib is a good treatment option for patients with pulmonary toxicities due to other TKIs, such as pleural effusion and pulmonary arterial hypertension, which have primarily been associated with dasatinib treatment [23, 37].

In particular, pulmonary arterial hypertension is very uncommon with bosutinib treatment in clinical trials, even after very long-term follow-up; however, there are a few case studies of patients experiencing pulmonary arterial hypertension when receiving bosutinib treatment following dasatinib or deterioration of pre-existing pulmonary arterial hypertension [38–42].

The risk of pleural effusion and discontinuation rates due to pleural effusion are low with bosutinib (Supplemental Table 1 and Supplemental Table 2, Online Resource 1). The annual incidence of pleural effusion remains relatively constant even during later years of treatment [20, 22]; the median time to first pleural effusion event was 626 days, and some events occurred 5–8 years after start of treatment [21]. It should be noted that the rate of pleural effusion with bosutinib is lower than with dasatinib, which is associated with a particularly high rate of pleural effusion (e.g. 1.9% with bosutinib in BFORE and 14% with dasatinib in DASISION) [13, 43]. Studies in patients who had received prior TKIs, including dasatinib, have shown that prior TKI treatment is not a risk factor for developing pleural effusion with bosutinib [21]. However, recurrent pleural effusion on bosutinib has been reported in 28–52% of patients who developed pleural effusion on dasatinib before switching [44–48], and therefore close monitoring is warranted.

Patients should be screened for risk factors for pleural effusion, and those at high-risk should be monitored (Table 1) [21]. Risk factors for pleural effusion with bosutinib have been identified as age ≥ 65 years, history of effusion events, history of hypertension, history of tobacco use, history of pulmonary events, and treatment with bosutinib or dasatinib [20, 21]. Regular screening for pleural effusion is not required for patients receiving bosutinib. However, patients should be monitored for symptoms of shortness of breath, particularly on exertion or orthopnea, or edema, and managed using supportive care, including diuresis or steroids, and dose modifications [1, 2, 23].

## Dosing strategies with bosutinib

**Fig. 1:**
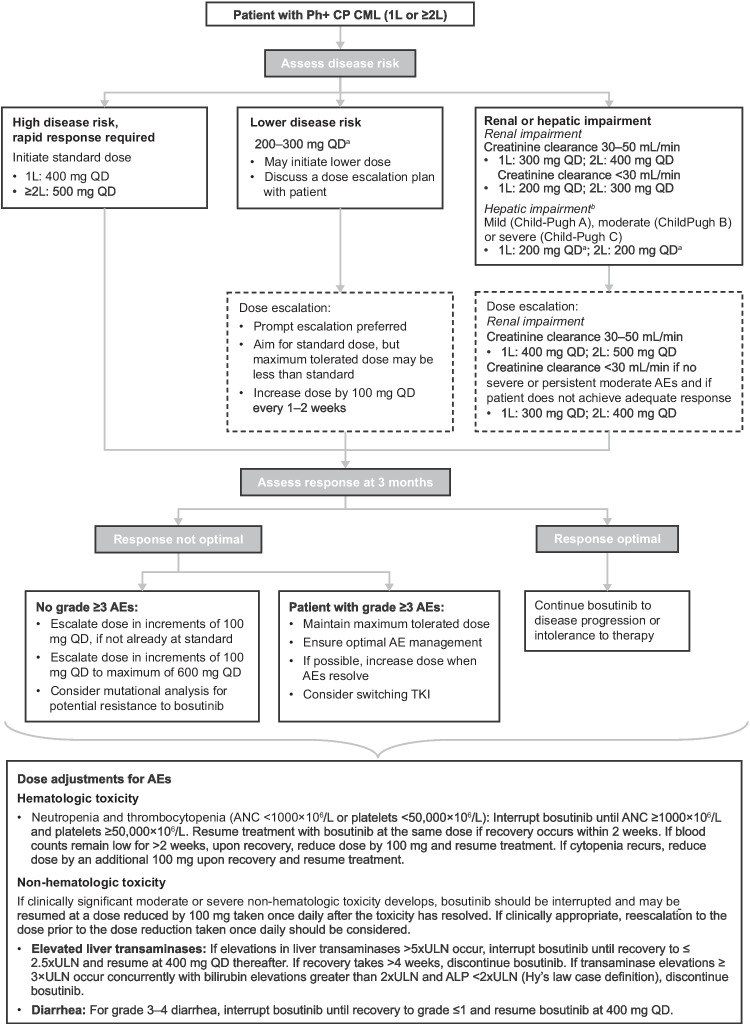
Dosing strategies for bosutinib therapy in CP-CML. Figure updated from Cortes et al. 2018 [8], including prescribing information [1, 2]. ^a^There are no clinical data for the efficacy of 200 mg QD bosutinib in CML. ^b^The European Medicines Agency lists hepatic impairment as a contraindication for bosutinib. Abbreviations: 1L, first line; 2L, second line; AEs, adverse events; ALP, alkaline phosphatase; ANC, absolute neutrophil count; CML, chronic myeloid leukemia; CP, chronic phase; TKI, tyrosine kinase inhibitor; ULN, upper limit of normal

The approved starting dose of bosutinib is 500 mg/day in the second- and later-line setting and 400 mg/day in the first-line setting [1, 2]. However, the minimum effective dose of bosutinib has not been established [8]. As shown in the dosing strategy algorithm in Fig. 1, adjusting the dose at the start of treatment or during treatment with bosutinib is an important strategy to manage AEs and improve tolerability, which are recognized within the label and in treatment guidelines [1, 2, 23, 24].

Certain patient populations, including older patients who are more likely to present with comorbidities, may particularly benefit from a personalized starting dose of bosutinib. For example, the bosutinib label includes recommendations of a lower starting dose in patients with moderate or severe renal or liver impairment, and the dose is escalated in the absence of AEs [1, 2]. In the BEST trial, a low starting dose of bosutinib in the second-line treatment of elderly patients with CML was highly effective and better tolerated than the approved 500 mg/day. Patients started on 200 mg/day and escalated to 300 mg/day after 2 weeks. Then, depending on response at 3 months, the dose could be further escalated to 400 mg/day for those not yet reaching *BCR*::*ABL1* < 1% at that timepoint. After 5 years, nearly 70% of patients remained on bosutinib, and nearly all were in major molecular response (MMR) [49]. The benefits of a lower bosutinib starting dose in older patients with comorbidities have been confirmed in real-world studies [34, 50]. In Japanese patients, a dose-escalation regimen (starting at 100 mg/day and increased by 100 mg every 2 weeks) improved tolerability versus starting at 500 mg/day and avoided treatment interruption and discontinuations due to AEs [51]. The BODO study evaluated a step-up dosing approach with bosutinib in patients with CML after failure or intolerance to second-generation TKIs. A starting dose of 300 mg/day bosutinib was increased in increments of 100 mg every 14 days in the absence of AEs to a maximum of 500 mg. While this approach was safe and efficacious, it was not associated with a reduction in the incidence of grade 2–4 toxicity, although the trial was stopped prematurely owing to slow recruitment after inclusion of 57 of 127 planned participants [52]. Nevertheless, bosutinib treatment showed notable efficacy with an MMR rate of 79% at Month 24; additionally, 64% of patients refractory to previous therapy and not in MMR at baseline achieved MMR during treatment [52].

Bosutinib dose reductions or interruptions in the event of an AE are associated with improved tolerability, enabling patients to continue treatment while maintaining efficacy (Table 2). Individual patient treatment goals can be adjusted on the basis of the need to balance safety and efficacy. In general, permanent doses below 300 mg are not recommended but could be used in individual instances for patients who already have a good, stable response. In BFORE, dose reductions from 400 mg/day bosutinib to either 300 mg or 200 mg were associated with a decrease in incidence of AEs, including diarrhea. These dose reductions enabled patients to stay on treatment and achieve improvement in outcomes [53]. After 5 years of follow up in BFORE, GI, liver, effusion, and renal treatment-emergent AEs were generally managed with dose adjustments. Successful treatment re-challenge was achieved in > 70% of patients with dose interruptions who were re-administered bosutinib [22]. In previously treated patients whose dose reduced from 500 mg/day, long-term efficacy was similar to that in patients who remained on 500 mg but with a lower incidence of AEs, particularly GI events [54]. Thus, dose reduction should be considered to manage AEs rather than immediate treatment discontinuation. For those patients where a temporary treatment interruption is required, most are successfully re-challenged without recurrence of events and/or treatment discontinuations [15]. The importance of bosutinib dose modifications for continued treatment and improved outcomes has been highlighted in the ASCEMBL trial, which evaluated asciminib (40 mg twice daily) versus bosutinib (500 mg once daily) in patients who had been treated with at least two prior TKIs [55]. In this trial, outcomes were significantly improved with asciminib versus bosutinib, with the 500-mg dose of bosutinib causing a high rate of discontinuations due to AEs. The treatment discontinuation rate of 21% due to AEs is 50% greater than in BEST (14%), even when the latter study had longer follow-up and included only older patients [56].

**Table 2:** Studies investigating bosutinib dosing strategies

Study (reference)	Patient population	Bosutinib dosing strategies	Key efficacy outcomes following described dosing strategy	Key safety outcomes
Phase 2 BODO study [52] (NCT02577926)	2L or 3L CP CML after failure or intolerance to 2G-TKIs (*N* = 57)	• Starting dose: 300 mg QD • Dose increases: increments of 100 mg daily every 14 days (absence of > G1 toxicities) • Maximum dose: 500 mg QD	Probability of MMR after 24 months: 79%	• ≥ 1 any grade TEAE: 100% • ≥ 1 grade 3/4 TEAE: 71.9% • SAEs: 28.1% • Most common AEs: ○ Diarrhea: 55.5% ○ Nausea: 16.2% ○ Abdominal pain: 9.8% ○ Increased ALT: 26.7% ○ Increased AST: 17.0%
Phase 1/2 BELA study [54] (NCT00261846)	Ph + CP CML resistant or intolerant to previous TKI therapy, AP CML, BP CML, or ALL (*n* = 570)	• Starting dose: 500 mg/day • Dose decreases to: ○ 400 mg/day (*n* = 144) ○ 300 mg/day (*n* = 95)	• 400 mg/day: ○ CCyR before and after dose reduction: 15% ○ CCyR after dose reduction: 28% ○ CCyR only before dose reduction: 3% • 300 mg/day: ○ CCyR before and after dose reduction: 24% ○ CCyR after dose reduction: 14% ○ CCyR only before dose reduction: 3%	• Incidence of TEAEs lower after dose reductions, particularly GI events • Incidence of hematological toxicities generally similar before and after dose reduction
Real-world study in patients > 65 years in Italy [50]	CP CML in ≥ 2L (*n* = 101)	• 500 mg/day: *n* = 25 • 400 mg/day: *n* = 7 • 300 mg/day: *n* = 33 • 200 mg/day: *n* = 34 • 100 mg/day: *n* = 2	• CCyR: 77.0% • MR: 66.7% • MMR: 21.8%	• Grade 3/4 hematological toxicity: 6.9% • Grade 3/4 extra-hematological toxicity: 18.8% • Permanent bosutinib discontinuation due to toxicity: 11.9%
Phase 3 BFORE trial dose reduction [53] (NCT02130557)	Newly diagnosed CP CML (bosutinib, *n* = 268)	• Starting dose: 400 mg QD • Dose reductions: decrements of 100 mg for toxicity • Minimum dose: 200 mg QD	• 300 mg: ○ MMR before and after dose reduction: 20.0% ○ MMR for first time after dose reduction: 45.0% • 200 mg: ○ MMR before and after dose reduction: 17.4% ○ MMR for first time after dose reduction: 17.4%	• 400 to 300 mg, > 10% decrease in AEs: ○ Diarrhea 71.3 to 36.3% ○ Nausea: 32.5 to 13.8% ○ Thrombocytopenia: 26.3 to 15.0% • 300 to 200 mg, > 10% decrease in AEs: ○ Diarrhea 82.6 to 21.7% ○ Nausea: 30.4 to 4.3% ○ Rash: 26.1 to 8.7% ○ Neutropenia: 13.0 to 4.3%
Phase 2 dose optimization study in 2L CML in elderly patients [49] (NCT02810990)	> 60 years CP CML intolerant or failed with 1L TKI (*n* = 63)	• Starting dose: 200 mg QD for 2 weeks • Dose increased to 300 mg QD for 3 months ○ Patients with BCR-ABLIS transcript ≤ 1% continued 300 mg QD ○ Patients with BCR-ABLIS transcript > 1% dose increased to 400 mg ○ Dose maintained at 300 mg/day or 400 mg/day in responsive patients	• MR3 at 1 year: 60% • MR4: 38% • MR4.5: 19%	• Selected AEs: ○ Cardiac ischemia, *n* = 2 ○ Pericardial effusion, *n* = 2 • AEs leading to permanent treatment discontinuation: ○ Hypertransaminasemia, *n* = 3 ○ Nephrotoxicity, *n* = 1 ○ Diarrhea, *n* = 1 ○ Skin rash: *n* = 1 ○ Myalgia/fatigue, *n* = 1
Phase 2 dose optimization study in 2L CML in elderly patients [57] (NCT02810990)	> 60 years CP CML intolerant or failed with 1L TKI (*n* = 63)	• Starting dose: 200 mg QD for 2 weeks • Dose increased to 300 mg QD for 3 months ○ Patients with BCR-ABLIS transcript ≤ 1% continued 300 mg QD ○ Patients with BCR-ABLIS transcript > 1% dose increased to 400 mg • Dose maintained at 300 mg/day or 400 mg/day in responsive patients	• MR3 at 1 year: 59% • After 36 months probability of: ○ MR3: 78% ○ MR4: 54% ○ MR4.5: 46% • OS: 81%	• CV AEs ○ Acute coronary syndromes, *n* = 6 ○ Pericarditis, *n* = 2 ○ Peripheral arterial thrombosis, *n* = 1 • AEs leading to permanent treatment discontinuation: ○ Transaminase increase, *n* = 5 ○ Skin rash, *n* = 1 ○ Myalgia, *n* = 1 ○ GI toxicity, *n* = 1 ○ Renal failure, *n* = 1
Standard dose vs dose escalation in Japanese CML patients [51]	CML (*n* = 25)	• Standard dosing group: 500 mg/day from start of treatment (*n* = 10) • Dose escalation group: starting at 100 mg/day and increasing by 100 mg/day every 2 weeks (*n* = 15)	N/A	• Patients with dose interruptions due to AEs: ○ Standard dosing: 90% ○ Dose escalation: 13.3% • Total duration of treatment interruption: ○ Standard dosing: 35 days ○ Dose escalation: 14 days • Median time to liver dysfunction ○ Standard dosing: 28 days ○ Dose escalation: 53.5 days • Median time to diarrhea ○ Standard dosing: 1 day ○ Dose escalation: 19 days

*1L* first line; *2L* second line; *3L* third line; *AE* adverse event; *ALL* acute lymphoblastic leukemia; *AP* accelerated phase; *AST* aspartate aminotransferase; *BP* blast phase; *CCyR* complete cytogenic response; *CML* chronic myeloid leukemia; *CP* chronic phase; *MMR* major molecular response; *MR* molecular response; *N/A* not available; *OS* overall survival; *Ph* + Philadelphia chromosome–positive; *QD* once daily; *TEAE* treatment emergent adverse event; *TKI* tyrosine kinase inhibitor

Treatment-free remission (TFR), with appropriate monitoring, may reduce the risk of AEs and can be considered in patients who meet the criteria that have been established for imatinib, dasatinib, or nilotinib in previous trials. It must be stated, however, that there are no prospective TFR studies reported with bosutinib, and TFR is not recommended in most patients who are treated with bosutinib (or other TKIs) in the later-line setting.

## Conclusions

Bosutinib is an effective and tolerable treatment option for patients with CML. Long-term results from clinical trials and emerging real-world evidence have demonstrated a manageable safety profile with treatment modifications and/or supportive care. Increased experience in managing toxicities and by using a personalized dosing approach may further improve adherence and outcomes with bosutinib.

### Supplementary Information

Below is the link to the electronic supplementary material.

Supplementary file1 (PDF 125 KB)

Supplementary file2 (DOCX 189 KB)

## Data Availability

No datasets were generated or analysed during the current study.

## References

[1] US Food and Drug Administration (2012) Bosulif (bosutinib) prescribing information. https://www.accessdata.fda.gov/drugsatfda_docs/label/2021/203341s020lbl.pdf. Accessed 8 Jan 2024

[2] European Medicines Agency (2013) Bosulif (bosutinib) summary of product characteristics. https://www.ema.europa.eu/en/documents/product-information/bosulif-epar-product-information_en.pdf. Accessed 8 Jan 2024

[3] Remsing Rix LL, Rix U, Colinge J et al (2009) Global target profile of the kinase inhibitor bosutinib in primary chronic myeloid leukemia cells. Leukemia 23:477–485. 10.1038/leu.2008.334 19039322 10.1038/leu.2008.334

[4] McClelland CM, Harocopos GJ, Custer PL (2010) Periorbital edema secondary to imatinib mesylate. Clin Ophthalmol 4:427–431. 10.2147/opth.s8521 20505834 10.2147/opth.s8521 PMC2874269

[5] Tsao AS, Kantarjian H, Cortes J et al (2003) Imatinib mesylate causes hypopigmentation in the skin. Cancer 98:2483–2487. 10.1002/cncr.11812 14635084 10.1002/cncr.11812

[6] Giona F, Mariani S, Gnessi L et al (2013) Bone metabolism, growth rate and pubertal development in children with chronic myeloid leukemia treated with imatinib during puberty. Haematologica 98:e25-27. 10.3324/haematol.2012.067447 22983586 10.3324/haematol.2012.067447 PMC3659938

[7] Lipton JH, Brümmendorf TH, Gambacorti-Passerini C et al (2022) Long-term safety review of tyrosine kinase inhibitors in chronic myeloid leukemia - What to look for when treatment-free remission is not an option. Blood Rev 56:100968. 10.1016/j.blre.2022.100968 35570071 10.1016/j.blre.2022.100968

[8] Cortes JE, Apperley JF, DeAngelo DJ et al (2018) Management of adverse events associated with bosutinib treatment of chronic-phase chronic myeloid leukemia: expert panel review. J Hematol Oncol 11:143. 10.1186/s13045-018-0685-2 30587215 10.1186/s13045-018-0685-2 PMC6307238

[9] Hochhaus A, Baccarani M, Silver RT et al (2020) European LeukemiaNet 2020 recommendations for treating chronic myeloid leukemia. Leukemia 34:966–984. 10.1038/s41375-020-0776-2 32127639 10.1038/s41375-020-0776-2 PMC7214240

[10] Smith G, Apperley J, Milojkovic D et al (2020) A British society for haematology guideline on the diagnosis and management of chronic myeloid leukaemia. Br J Haematol 191:171–193. 10.1111/bjh.16971 32734668 10.1111/bjh.16971

[11] Cortes JE, Kantarjian HM, Brümmendorf TH et al (2011) Safety and efficacy of bosutinib (SKI-606) in chronic phase Philadelphia chromosome-positive chronic myeloid leukemia patients with resistance or intolerance to imatinib. Blood 118:4567–4576. 10.1182/blood-2011-05-355594 21865346 10.1182/blood-2011-05-355594 PMC4916618

[12] Khoury HJ, Cortes JE, Kantarjian HM et al (2012) Bosutinib is active in chronic phase chronic myeloid leukemia after imatinib and dasatinib and/or nilotinib therapy failure. Blood 119:3403–3412. 10.1182/blood-2011-11-390120 22371878 10.1182/blood-2011-11-390120 PMC4916559

[13] Cortes JE, Gambacorti-Passerini C, Deininger MW et al (2018) Bosutinib versus imatinib for newly diagnosed chronic myeloid leukemia: Results from the randomized BFORE trial. J Clin Oncol 36:231–237. 10.1200/JCO.2017.74.7162 29091516 10.1200/JCO.2017.74.7162 PMC5966023

[14] Cortes JE, Kim D-W, Kantarjian HM et al (2012) Bosutinib versus imatinib in newly diagnosed chronic-phase chronic myeloid leukemia: results from the BELA trial. J Clin Oncol 30:3486–3492. 10.1200/JCO.2011.38.7522 22949154 10.1200/JCO.2011.38.7522 PMC4979199

[15] Gambacorti-Passerini C, Cortes JE, Lipton JH et al (2014) Safety of bosutinib versus imatinib in the phase 3 BELA trial in newly diagnosed chronic phase chronic myeloid leukemia. Am J Hematol 89:947–953. 10.1002/ajh.23788 24944159 10.1002/ajh.23788 PMC4305212

[16] Gambacorti-Passerini C, Cortes JE, Lipton JH et al (2018) Safety and efficacy of second-line bosutinib for chronic phase chronic myeloid leukemia over a five-year period: final results of a phase I/II study. Haematologica 103:1298–1307. 10.3324/haematol.2017.171249 29773593 10.3324/haematol.2017.171249 PMC6068045

[17] Gambacorti-Passerini C (2021) Second-line bosutinib (BOS) for patients (pts) with chronic phase (CP) chronic myeloid leukemia (CML): Final 10-year results of a phase 1/2 study. J Clin Oncol 39:Abstract-7009.

[18] Hochhaus A, Gambacorti-Passerini C, Abboud C et al (2020) Bosutinib for pretreated patients with chronic phase chronic myeloid leukemia: primary results of the phase 4 BYOND study. Leukemia 34:2125–2137. 10.1038/s41375-020-0915-9 32572189 10.1038/s41375-020-0915-9 PMC7387243

[19] Brümmendorf TH, Cortes JE, Goh YT et al (2020) Bosutinib (BOS) for chronic phase (CP) chronic myeloid leukemia (CML) after imatinib (IMA) failure: ≥8-y update of a phase I/II study. JCO 38(Suppl 15):7549. 10.1200/JCO.2020.38.15_suppl.7549 10.1200/JCO.2020.38.15_suppl.7549

[20] Brümmendorf TH, Cortes JE, Milojkovic D et al (2022) Bosutinib versus imatinib for newly diagnosed chronic phase chronic myeloid leukemia: final results from the BFORE trial. Leukemia 36:1825–1833. 10.1038/s41375-022-01589-y 35643868 10.1038/s41375-022-01589-y PMC9252917

[21] Cortes JE, Kantarjian HM, Mauro MJ et al (2021) Long-term cardiac, vascular, hypertension, and effusion safety of bosutinib in patients with Philadelphia chromosome-positive leukemia resistant or intolerant to prior therapy. Eur J Haematol 106:808–820. 10.1111/ejh.13608 33638218 10.1111/ejh.13608

[22] Cortes JE, Milojkovic D, Gambacorti-Passerini C et al (2022) Bosutinib (BOS) in newly diagnosed chronic myeloid leukemia (CML): gastrointestinal (GI), liver, effusion, and renal safety characterization in the BFORE trial. JCO 40(Suppl 16):7049. 10.1200/JCO.2022.40.16_suppl.7049 10.1200/JCO.2022.40.16_suppl.7049

[23] Steegmann JL, Baccarani M, Breccia M et al (2016) European LeukemiaNet recommendations for the management and avoidance of adverse events of treatment in chronic myeloid leukaemia. Leukemia 30:1648–1671. 10.1038/leu.2016.104 27121688 10.1038/leu.2016.104 PMC4991363

[24] National Comprehensive Cancer Network (2023) NCCN Guidelines. CML. Version 1. https://www.nccn.org/guidelines/guidelines-detail. Accessed 8 Jan 2024

[25] Milojkovic D, Lyon AR, Mehta P et al (2023) Cardiovascular risk in chronic myeloid leukaemia: a multidisciplinary consensus on screening and management. Eur J Haematol 111:201–210. 10.1111/ejh.13983 37186398 10.1111/ejh.13983

[26] Gambacorti-Passerini C, le Coutre P, Piazza R (2020) The role of bosutinib in the treatment of chronic myeloid leukemia. Future Oncol 16:4395–4408. 10.2217/fon-2019-0555 31833784 10.2217/fon-2019-0555

[27] Cortes JE, Gambacorti-Passerini C, Kim D-W et al (2017) Effects of bosutinib treatment on renal function in patients with Philadelphia chromosome-positive leukemias. Clin Lymphoma Myeloma Leuk 17:684-695.e686. 10.1016/j.clml.2017.06.001 28807791 10.1016/j.clml.2017.06.001

[28] Brummendorf TH, Cortes JE, de Souza CA et al (2015) Bosutinib versus imatinib in newly diagnosed chronic-phase chronic myeloid leukaemia: results from the 24-month follow-up of the BELA trial. Br J Haematol 168:69–81. 10.1111/bjh.13108 25196702 10.1111/bjh.13108 PMC4274978

[29] Cortes JE, le Coutre PD, Gambacorti-Passerini C et al (2020) Long-term cardiac, vascular, and hypertension safety of bosutinib (BOS) versus imatinib (IMA) for newly diagnosed chronic myeloid leukemia (CML): Results from the BFORE trial. Blood 136:34–35. 10.1182/blood-2020-134912 10.1182/blood-2020-134912

[30] Khoury HJ, Gambacorti-Passerini C, Brümmendorf TH (2018) Practical management of toxicities associated with bosutinib in patients with Philadelphia chromosome-positive chronic myeloid leukemia. Ann Oncol 29:578–587. 10.1093/annonc/mdy019 29385394 10.1093/annonc/mdy019 PMC5888919

[31] Vidal-Petiot E, Rea D, Serrano F et al (2016) Imatinib increases serum creatinine by inhibiting its tubular secretion in a reversible fashion in chronic myeloid leukemia. Clin Lymphoma Myeloma Leuk 16:169–174. 10.1016/j.clml.2015.12.001 26795084 10.1016/j.clml.2015.12.001

[32] Omote S, Matsuoka N, Arakawa H et al (2018) Effect of tyrosine kinase inhibitors on renal handling of creatinine by MATE1. Sci Rep 8:9237. 10.1038/s41598-018-27672-y 29915248 10.1038/s41598-018-27672-y PMC6006426

[33] Abumiya M, Takahashi N, Takahashi S et al (2021) Effects of SLC22A2 808G>T polymorphism and bosutinib concentrations on serum creatinine in patients with chronic myeloid leukemia receiving bosutinib therapy. Sci Rep 11:6362. 10.1038/s41598-021-85757-7 33737618 10.1038/s41598-021-85757-7 PMC7973796

[34] Claudiani S, Janssen JJWM, Byrne J et al (2022) A retrospective observational research study to describe the real-world use of bosutinib in patients with chronic myeloid leukemia in the United Kingdom and the Netherlands. Eur J Haematol 109:90–99. 10.1111/ejh.13775 35403752 10.1111/ejh.13775 PMC9321569

[35] Brümmendorf TH, Cortes JE, Busque L et al (2021) The effect of body mass index on efficacy and safety of bosutinib or imatinib in patients with newly diagnosed chronic myeloid leukemia. JCO 39(Suppl 15):7037. 10.1200/JCO.2021.39.15_suppl.7037 10.1200/JCO.2021.39.15_suppl.7037

[36] Brümmendorf TH, Mamolo CM, Reisman A et al (2018) Impact of diarrhea on health-related quality of life: analysis of the phase 3 BFORE trial of bosutinib vs imatinib for newly diagnosed chronic phase chronic myeloid leukemia. Blood 132(Suppl 1):4264–4264. 10.1182/blood-2018-99-110152 10.1182/blood-2018-99-110152

[37] Cortes J (2020) How to manage CML patients with comorbidities. Blood 136:2507–2512. 10.1182/blood.2020006911 33236757 10.1182/blood.2020006911

[38] Hickey PM, Thompson AA, Charalampopoulos A et al (2016) Bosutinib therapy resulting in severe deterioration of pre-existing pulmonary arterial hypertension. Eur Respir J 48:1514–1516. 10.1183/13993003.01004-2016 27660511 10.1183/13993003.01004-2016

[39] Parthvi R, Gibbons W (2020) Pulmonary arterial hypertension worsened by bosutinib in patient with previous dasatinib treatment. Am J Ther 28:e704–e706. 10.1097/mjt.0000000000001156 32947343 10.1097/mjt.0000000000001156

[40] Seegobin K, Babbar A, Ferreira J et al (2017) A case of worsening pulmonary arterial hypertension and pleural effusions by bosutinib after prior treatment with dasatinib. Pulm Circ 7:808–812. 10.1177/2045893217733444 28914582 10.1177/2045893217733444 PMC5703128

[41] Takatsuka I, Hirata H, Takahashi T et al (2022) Successful treatment with nilotinib after bosutinib-induced pulmonary arterial hypertension recurrence following dasatinib in chronic myeloid leukemia in chronic phase. Leuk Res Rep 17:100312. 10.1016/j.lrr.2022.100312 35509967 10.1016/j.lrr.2022.100312 PMC9059076

[42] Yo S, Thenganatt J, Lipton J et al (2020) Incident pulmonary arterial hypertension associated with Bosutinib. Pulm Circ 10:2045894020936913. 10.1177/2045894020936913 32913629 10.1177/2045894020936913 PMC7443988

[43] Kantarjian HM, Shah NP, Cortes JE et al (2012) Dasatinib or imatinib in newly diagnosed chronic-phase chronic myeloid leukemia: 2-year follow-up from a randomized phase 3 trial (DASISION). Blood 119:1123–1129. 10.1182/blood-2011-08-376087 22160483 10.1182/blood-2011-08-376087 PMC4916556

[44] Aslan NA, Hıncal HO, Elver Ö et al (2023) Bosutinib-induced massive pleural effusion: Cross-intolerance with all tyrosine kinase inhibitors. J Oncol Pharm Pract 29:511–516. 10.1177/10781552221114070 35821583 10.1177/10781552221114070

[45] Cortes JE, Lipton JH, Kota V et al (2023) Cross-intolerance with bosutinib after prior tyrosine kinase inhibitors for Philadelphia chromosome-positive leukemia: long-term analysis of a phase I/II study. Haematologica 108:3454–3459. 10.3324/haematol.2022.281944 37439348 10.3324/haematol.2022.281944 PMC10690913

[46] García-Gutiérrez V, Milojkovic D, Hernandez-Boluda JC et al (2019) Safety and efficacy of bosutinib in fourth-line therapy of chronic myeloid leukemia patients. Ann Hematol 98:321–330. 10.1007/s00277-018-3507-2 30446802 10.1007/s00277-018-3507-2

[47] Jain AG, Gesiotto Q, Ball S et al (2024) Incidence of pleural effusion with dasatinib and the effect of switching therapy to a different TKI in patients with chronic phase CML. Ann Hematol 103:1941–1945. 10.1007/s00277-024-05760-6 38634915 10.1007/s00277-024-05760-6 PMC11090947

[48] Riou M, Seferian A, Savale L et al (2016) Deterioration of pulmonary hypertension and pleural effusion with bosutinib following dasatinib lung toxicity. Eur Respir J 48:1517–1519. 10.1183/13993003.01410-2016 27799395 10.1183/13993003.01410-2016

[49] Castagnetti F, Gugliotta G, Bocchia M et al (2019) Dose optimization in elderly CML patients treated with bosutinib after intolerance or failure of first-line tyrosine kinase inhibitors. Blood 134(Suppl 1):496. 10.1182/blood-2019-12751431395581 10.1182/blood-2019-127514

[50] Latagliata R, Attolico I, Trawinska MM et al (2021) Bosutinib in the real-life treatment of chronic myeloid leukemia patients aged >65 years resistant/intolerant to previous tyrosine-kinase inhibitors. Hematol Oncol 39:401–408. 10.1002/hon.2851 33617659 10.1002/hon.2851

[51] Mita A, Abumiya M, Miura M et al (2018) Correlation of plasma concentration and adverse effects of bosutinib: standard dose or dose-escalation regimens of bosutinib treatment for patients with chronic myeloid leukemia. Exp Hematol Oncol 7:9. 10.1186/s40164-018-0101-1 29682402 10.1186/s40164-018-0101-1 PMC5899348

[52] Isfort S, Wolf D, Teichmann LL et al (2021) Step-in dosing in the Bosutinib dose optimization study (BODO) failed to reduce gastrointestinal (GI) toxicity in patients failing second generation TKI (2G-TKI) in chronic phase chronic myeloid leukemia (CML) but suggests promising molecular response. Blood 138(Suppl 1):3608. 10.1182/blood-2021-151240 10.1182/blood-2021-151240

[53] Brummendorf TH, Gambacorti-Passerini C, Hochhaus A et al (2018) Efficacy and safety following dose reduction of bosutinib or imatinib in patients with newly diagnosed chronic myeloid leukemia: analysis of the phase 3 BFORE trial. Blood 132(Suppl 1):3005. 10.1182/blood-2018-99-110543 10.1182/blood-2018-99-110543

[54] Kota V, Brümmendorf TH, Gambacorti-Passerini C et al (2021) Efficacy and safety following bosutinib dose reduction in patients with Philadelphia chromosome-positive leukemias. Leuk Res 111:106690. 10.1016/j.leukres.2021.106690 34673442 10.1016/j.leukres.2021.106690

[55] Réa D, Mauro MJ, Boquimpani C et al (2021) A phase 3, open-label, randomized study of asciminib, a STAMP inhibitor, vs bosutinib in CML after 2 or more prior TKIs. Blood 138:2031–2041. 10.1182/blood.2020009984 34407542 10.1182/blood.2020009984 PMC9728405

[56] Hochhaus A, Réa D, Boquimpani C et al (2023) Asciminib vs bosutinib in chronic-phase chronic myeloid leukemia previously treated with at least two tyrosine kinase inhibitors: longer-term follow-up of ASCEMBL. Leukemia 37:617–626. 10.1038/s41375-023-01829-9 36717654 10.1038/s41375-023-01829-9 PMC9991909

[57] Castagnetti F, Bocchia M, Abruzzese E et al (2022) P698: Bosutinib dose optimization in the second-line treatment of elderly cml patients: extended 3-year follow-up and final results of the best study. HemaSphere 6:593. 10.1097/01.HS9.0000845676.81208.c2 10.1097/01.HS9.0000845676.81208.c2

