# Supplementary material for: Practical considerations in the management of patients treated with bosutinib for chronic myeloid leukemia
Source: Ann Hematol. 2024 Jul 18;103(9):3429–42. doi: 10.1007/s00277-024-05851-4 (PMC11358173; doi:10.1007/s00277-024-05851-4)
Supplement: Supplementary file 1 — Supplementary file1 (PDF 125 KB) [file 277_2024_5851_MOESM1_ESM.pdf]

Plain Language Summary

Please note that this summary only contains information from the full scientific article:

View Scientific Article

A summary on preventing and managing side effects of bosutinib for people with chronic myeloid leukemia

Alanine aminotransferase
<A-luh-neen uh-MEE-noh-TRANZ-feh-race>

Aspartate aminotransferase
<as-PAR-tayt uh-MEE-noh-TRANZ-feh-race>

Bosutinib
<boh-SOO-tih-nib>

Chromosome
<KROH-muh-some>

Chronic myeloid leukemia
<KRAH-nik MY-eh-loyd loo-KEE-mee-uh>

Dasatinib
<da-SA-tih-nib>

Electrolyte
<ee-LEK-troh-lite>

Electrocardiogram
<ee-LEK-troh-KAR-dee-oh-gram>

Imatinib
<ih-MA-tih-nib>

Neutrophil
<NOO-troh-fil>

Nilotinib
<nye-LOH-tih-nib>

Platelet
<PLAYT-let>

Tyrosine kinase inhibitor
<TY-ruh-seen KY-nays in-HIH-bih-ter>

Additional information

More information can be found in the scientific article of this study, which you can access here:

View Scientific Article

Date of summary: tbd 2023

The full title of this article: Practical considerations in the management of patients treated with bosutinib for chronic myeloid leukemia.

Key takeaways

- The review provides practical recommendations for how to stop and manage side effects experienced by some people taking bosutinib.
- There is some doable advice your doctor can give you to stop side effects. They can also advise you to identify the signs and symptoms.
- This review may help doctors treating people with CML taking bosutinib manage their side effects and continue bosutinib treatment.

The purpose of this plain language summary is to help you to understand the findings from recent research.

- Bosutinib is approved to treat chronic myeloid leukemia.
- Researchers must look at the results of many types of studies to understand whether a study drug works, how it works, and whether it is safe to prescribe to patients.
- This summary reports information from a review article looking at results from several studies. The findings might be different from other review articles depending on how the studies included were done or other information needed to develop the review.

What did this review look at?

- This review looked at useful advice for patients and doctors about how to prevent and manage possible side effects experienced when taking bosutinib.

What is chronic myeloid leukemia?

- Chronic myeloid leukemia (CML) is a type of cancer that affects white blood cells.
  - Leukemia cells are abnormal white blood cells.
  - Chronic means that it tends to grow slowly over many years.
- CML is caused by the formation of an abnormal fusion gene called BCR-ABL1.
  - A fusion gene is made by joining parts of 2 different genes into a completely new gene.
    - Genes are parts of DNA that are found in structures called chromosomes. They are found in every cell of the body.
    - DNA is a molecule in a person's cells that tells the cells how to work.
    - Chromosomes are bundles of DNA.
    - The BCR-ABL1 gene is an example of a fusion gene.
- The BCR-ABL1 gene is found in the Philadelphia chromosome. It is present in some types of leukemia cancer cells and absent in healthy cells.

What is bosutinib?

- Bosutinib is a type of medicine known as a tyrosine kinase inhibitor (TKI).
  - Tyrosine kinases are proteins in the body that manage how cells grow and divide.
  - The BCR-ABL1 gene makes a tyrosine kinase that is more active than normal. This makes leukemia cells grow faster than healthy cells.
  - Bosutinib works by blocking this more active tyrosine kinase in the leukemia cells, causing those cells to die.
- Bosutinib is taken by mouth daily.
- Bosutinib is an approved treatment for people who have:
  - CML who have not yet received treatment with a TKI (called newly diagnosed CML).
  - CML that is not responding to treatment with other TKIs (such as imatinib, dasatinib, and nilotinib) any longer.
  - Stopped their earlier treatment because they could not tolerate side effects.

Alanine aminotransferase
<A-luh-neen uh-MEE-noh-TRANZ-feh-race>

Aspartate aminotransferase
<as-PAR-tayt uh-MEE-noh-TRANZ-feh-race>

Bosutinib
<boh-SOO-tih-nib>

Chromosome
<KROH-muh-some>

Chronic myeloid leukemia
<KRAH-nik MY-eh-loyd loo-KEE-mee-uh>

Dasatinib
<da-SA-tih-nib>

Electrolyte
<ee-LEK-troh-lite>

Electrocardiogram
<ee-LEK-troh-KAR-dee-oh-gram>

Imatinib
<ih-MA-tih-nib>

Neutrophil
<NOO-troh-fil>

Nilotinib
<nye-LOH-tih-nib>

Platelet
<PLAYT-let>

Tyrosine kinase inhibitor
<TY-ruh-seen KY-nays in-HIH-bih-ter>

Additional information

More information can be found in the scientific article of this study, which you can access here:

View Scientific Article

Alanine aminotransferase
<A-luh-neen uh-MEE-noh-TRANZ-feh-race>

Aspartate aminotransferase
<as-PAR-tayt uh-MEE-noh-TRANZ-feh-race>

Bosutinib
<boh-SOO-tih-nib>

Chromosome
<KROH-muh-some>

Chronic myeloid leukemia
<KRAH-nik MY-eh-loyd loo-KEE-mee-uh>

Dasatinib
<da-SA-tih-nib>

Electrolyte
<ee-LEK-troh-lite>

Electrocardiogram
<ee-LEK-troh-KAR-dee-oh-gram>

Imatinib
<ih-MA-tih-nib>

Neutrophil
<NOO-troh-fil>

Nilotinib
<nye-LOH-tih-nib>

Platelet
<PLAYT-let>

Tyrosine kinase inhibitor
<TY-ruh-seen KY-nays in-HIH-bih-ter>

Additional information

More information can be found in the scientific article of this study, which you can access here:

View Scientific Article

What was the aim of this review?

- The review looked at information from several clinical and real-world studies of people taking bosutinib for CML. The writers of the review are doctors treating people with CML who are taking bosutinib.
- The review focused on the side effects that some people have experienced when receiving bosutinib. It gives practical advice about how doctors can stop or manage the side effects.
  - A side effect is something (expected or unexpected) that you feel was caused by a medicine or treatment you take.
- The authors looked at side effects affecting blood cells, side effects experienced by people taking all TKI medications (affecting the heart, veins, arteries, kidneys, and skin), and side effects experienced by people taking bosutinib (affecting the stomach and intestines, liver, and build-up of fluid around the lungs).
- This summary describes practical advice from the writers of the review about how to stop and manage specific side effects experienced by some people when taking bosutinib. It also includes how to adjust the amount of bosutinib.

What practical advice was given in the review?

Suggestions for preventing and managing side effects

Blood cells

- **Side effects:**
  Low levels of some blood cells are common and can occur early during bosutinib treatment, usually within the first year. Examples include:
  - Low levels of platelets, which help stop bleeding
  - Low levels of neutrophils, a type of white blood cell that helps fight infections.

What can you do?

- Look out for the signs and symptoms of these types of side effects:
  - Unexpected bruising
  - Fever and signs of infection
  - Unexpected bleeding
  - Blood in urine or blood in stool.
- Tell your doctor as soon as possible if you experience these types of side effects.

What can your doctor do?

- Regular blood tests to monitor changes in your blood cells
- If these side effects occur:
  - Lower the dose of bosutinib or pause bosutinib treatment until the levels of blood cells return to normal.
  - Keep the dose of bosutinib low if levels of blood cells stay low.
  - Treat the low levels of blood cells with other medications.
  - Some people will need to stop taking bosutinib.

Alanine aminotransferase
<A-luh-neen uh-MEE-noh-TRANZ-feh-race>

Aspartate aminotransferase
<as-PAR-tayt uh-MEE-noh-TRANZ-feh-race>

Bosutinib
<boh-SOO-tih-nib>

Chromosome
<KROH-muh-some>

Chronic myeloid leukemia
<KRAH-nik MY-eh-loyd loo-KEE-mee-uh>

Dasatinib
<da-SA-tih-nib>

Electrolyte
<ee-LEK-troh-lite>

Electrocardiogram
<ee-LEK-troh-KAR-dee-oh-gram>

Imatinib
<ih-MA-tih-nib>

Neutrophil
<NOO-troh-fil>

Nilotinib
<nye-LOH-tih-nib>

Platelet
<PLAYT-let>

Tyrosine kinase inhibitor
<TY-ruh-seen KY-nays in-HIH-bih-ter>

Additional information

More information can be found in the scientific article of this study, which you can access here:

View Scientific Article

Heart, veins, and arteries

- **Side effects:**
  Changes that affect the beating of the heart and/or circulation of blood

What can you do?

- Tell your doctor before starting bosutinib treatment if you have conditions affecting your heart or blood circulation.
- Talk to your doctor about possible lifestyle changes that might lower your risk factors for conditions affecting the heart and blood circulation.
- Look out for the signs and symptoms of the following side effects:
  - Fast, slow, or irregular heartbeat
  - Chest pain
  - High blood pressure (signs like headaches, nosebleeds, or fast heartbeat).
- Tell your doctor as soon as possible if you experience these types of side effects.

What can your doctor do?

- Assess and manage your individual risk factors for conditions affecting the heart and blood circulation.
- Speak with a cardiologist for more guidance for people with existing conditions affecting the heart and blood circulation or people at high risk of developing these types of side effects.
- Regular blood tests to monitor levels of electrolytes
  - Electrolytes are salts and minerals found in the blood that conduct electrical impulses in the body.
- Regular electrocardiograms for people with certain risk factors or conditions
  - An electrocardiogram checks your heart's rhythm and electrical activity.
- Look at the other medications that you take and how they might affect your heart and blood circulation and how they might change the way bosutinib works.
  - Some medications that you take might need to be adjusted or changed when you start bosutinib treatment.
- Start some people on a lower dose of bosutinib.
- If these side effects occur:
  - Look at all factors that might affect how your heart and blood circulation work, including other medications and your personal risk factors and lifestyle.
  - Lower the dose of bosutinib or pause bosutinib treatment until there are no more side effects.
  - Treat the side effects affecting the heart and blood circulation with other medications.
  - Some people will need to stop taking bosutinib.

Alanine aminotransferase
<A-luh-neen uh-MEE-noh-TRANZ-feh-race>

Aspartate aminotransferase
<as-PAR-tayt uh-MEE-noh-TRANZ-feh-race>

Bosutinib
<boh-SOO-tih-nib>

Chromosome
<KROH-muh-some>

Chronic myeloid leukemia
<KRAH-nik MY-eh-loyd loo-KEE-mee-uh>

Dasatinib
<da-SA-tih-nib>

Electrolyte
<ee-LEK-troh-lite>

Electrocardiogram
<ee-LEK-troh-KAR-dee-oh-gram>

Imatinib
<ih-MA-tih-nib>

Neutrophil
<NOO-troh-fil>

Nilotinib
<nye-LOH-tih-nib>

Platelet
<PLAYT-let>

Tyrosine kinase inhibitor
<TY-ruh-seen KY-nays in-HIH-bih-ter>

Kidneys

- **Side effects:**
  Changes that affect how well your kidneys work

What can you do?

- Tell your doctor before starting bosutinib treatment if you have conditions affecting your kidney.s
- Look out for the signs and symptoms of the following side effects:
  - Needing to urinate more often than usual.
  - Urinating less often than usual.
- Keep looking out for these signs and symptoms even if you have been taking bosutinib for a long time.
- Tell your doctor as soon as possible if you experience these side effects.

What can your doctor do?

- Test blood levels of creatinine before starting bosutinib treatment.
  - Creatinine is a waste product that is released into the blood by muscles and is cleared out by the kidneys.
  - How much creatinine is in your blood tells the doctor how well your kidneys are working.
- Start people on a lower dose of bosutinib if their kidneys are not working as well as they should.
- Continue regular blood tests of creatinine levels.
- If these side effects occur:
  - Look at all factors that might affect how your kidneys work, including other medications and your personal risk factors.
  - Lower the dose of bosutinib or pause bosutinib treatment until there are no more side effects.
  - Treat the side effects affecting the kidneys with other medications.
  - Some people will need to stop taking bosutinib.

Additional information

More information can be found in the scientific article of this study, which you can access here:

View Scientific Article

Alanine aminotransferase
<A-luh-neen uh-MEE-noh-TRANZ-feh-race>

Aspartate aminotransferase
<as-PAR-tayt uh-MEE-noh-TRANZ-feh-race>

Bosutinib
<boh-SOO-tih-nib>

Chromosome
<KROH-muh-some>

Chronic myeloid leukemia
<KRAH-nik MY-eh-loyd loo-KEE-mee-uh>

Dasatinib
<da-SA-tih-nib>

Electrolyte
<ee-LEK-troh-lite>

Electrocardiogram
<ee-LEK-troh-KAR-dee-oh-gram>

Imatinib
<ih-MA-tih-nib>

Neutrophil
<NOO-troh-fil>

Nilotinib
<nye-LOH-tih-nib>

Platelet
<PLAYT-let>

Tyrosine kinase inhibitor
<TY-ruh-seen KY-nays in-HIH-bih-ter>

Skin

- **Side effects:**
  Changes that affect how your skin feels and looks

What can you do?

- Tell your doctor before starting bosutinib treatment if you have conditions affecting your skin.
- Look out for the signs and symptoms of the following side effects:
  - Rash
  - Itching
  - Acne.
- Tell your doctor as soon as possible if you experience these types of side effects.
- Keep your skin as healthy as possible:
  - Avoid long, hot baths
  - Drink plenty of water.

What can your doctor do?

- If these side effects occur:
  - Treat the side effects affecting the skin with creams, ointments, or other medications.
  - Lower the dose of bosutinib or pause bosutinib treatment until recovery of side effects.
  - Some people will need to stop taking bosutinib.

Additional information

More information can be found in the scientific article of this study, which you can access here:

View Scientific Article

Alanine aminotransferase
<A-luh-neen uh-MEE-noh-TRANZ-feh-race>

Aspartate aminotransferase
<as-PAR-tayt uh-MEE-noh-TRANZ-feh-race>

Bosutinib
<boh-SOO-tih-nib>

Chromosome
<KROH-muh-some>

Chronic myeloid leukemia
<KRAH-nik MY-eh-loyd loo-KEE-mee-uh>

Dasatinib
<da-SA-tih-nib>

Electrolyte
<ee-LEK-troh-lite>

Electrocardiogram
<ee-LEK-troh-KAR-dee-oh-gram>

Imatinib
<ih-MA-tih-nib>

Neutrophil
<NOO-troh-fil>

Nilotinib
<nye-LOH-tih-nib>

Platelet
<PLAYT-let>

Tyrosine kinase inhibitor
<TY-ruh-seen KY-nays in-HIH-bih-ter>

Additional information

More information can be found in the scientific article of this study, which you can access here:

View Scientific Article

Stomach and intestines

- **Side effects:**
  Changes that affect how your stomach and intestines work and how you feel are very common, especially when you first start taking bosutinib

What can you do?

- Always take bosutinib tablets with food and water.
- Drink plenty of water.
- Avoid foods that might make you feel worse.
- Look out for the signs and symptoms of the following side effects, even from the first days after starting bosutinib treatment:
  - Diarrhea
  - Nausea
  - Vomiting
  - Pain in the stomach.
- Tell your doctor as soon as possible if you experience these types of side effects.

What can your doctor do?

- Check on how you're feeling, especially early in treatment, so they can help you manage these symptoms as soon as possible.
- If these side effects occur:
  - Treat the side effects with other medications, like anti-nausea or anti-diarrheal medications, and fluid replacement and advise on how best to take these medications while on bosutinib treatment.
  - Lower the dose of bosutinib or pause bosutinib treatment until there are no more side effects.
  - Some people will need to stop taking bosutinib.

Alanine aminotransferase
<A-luh-neen uh-MEE-noh-TRANZ-feh-race>

Aspartate aminotransferase
<as-PAR-tayt uh-MEE-noh-TRANZ-feh-race>

Bosutinib
<boh-SOO-tih-nib>

Chromosome
<KROH-muh-some>

Chronic myeloid leukemia
<KRAH-nik MY-eh-loyd loo-KEE-mee-uh>

Dasatinib
<da-SA-tih-nib>

Electrolyte
<ee-LEK-troh-lite>

Electrocardiogram
<ee-LEK-troh-KAR-dee-oh-gram>

Imatinib
<ih-MA-tih-nib>

Neutrophil
<NOO-troh-fil>

Nilotinib
<nye-LOH-tih-nib>

Platelet
<PLAYT-let>

Tyrosine kinase inhibitor
<TY-ruh-seen KY-nays in-HIH-bih-ter>

Additional information

More information can be found in the scientific article of this study, which you can access here:

View Scientific Article

Liver

- **Side effects:**
  Changes that affect how well your liver works, especially in the first few months of bosutinib treatment

What can you do?

- Tell your doctor before starting bosutinib treatment if you have conditions affecting your liver.
- Look out for the signs and symptoms of the following side effects:
  - Jaundice (your skin and the whites of your eyes turn yellow)
  - Dark urine
  - Pale stools
  - Itchy skin.
- Tell your doctor as soon as possible if you experience these types of side effects.
- Avoid having a lot of alcohol.

What can your doctor do?

- Test blood levels of alanine aminotransferase (ALT for short) and aspartate aminotransferase (AST for short) before starting bosutinib treatment.
  - ALT and AST are enzymes made by the liver.
  - The liver releases these enzymes into the blood when it is damaged.
  - Measuring the levels of liver enzymes in the blood tells the doctor how well your liver is working.
- Start people on a lower dose of bosutinib if their liver is not working as well as it should.
  - Some people might not be able to start bosutinib treatment if their liver enzyme levels in the blood are very high.
- Continue regular blood tests of liver enzyme levels, particularly in the first 3 months of bosutinib treatment.
- If these side effects occur:
  - Look at all factors that might affect how your liver works, including infections, other medications, and alcohol use.
  - Lower the dose of bosutinib or pause bosutinib treatment until recovery of side effects.
  - There are no other medications that will treat these side effects, but the doctor will advise about avoiding alcohol and other medications and supplements that might harm the liver.
  - Some people will need to stop taking bosutinib.

Lungs

- **Side effects:**
  A build-up of fluid in the space around your lungs

What can you do?

- Tell your doctor before starting bosutinib treatment if you have experienced previous build-up of fluid in the lungs or other diseases of the lungs.
- Look out for the signs and symptoms of the following side effects:
  - Shortness of breath, particularly when exercising or lying down
  - Coughing
  - Pain in the chest.
- Tell your doctor as soon as possible if you experience these types of side effects.

What can your doctor do?

- Assess and manage your individual risk factors for these types of side effects.
- If these side effects occur:
  - Drain out some of the fluid
  - Treat the side effects with other medications
  - Lower the dose of bosutinib or pause bosutinib treatment until there are no more side effects
  - Some people will need to stop taking bosutinib.

Alanine aminotransferase
<A-luh-neen uh-MEE-noh-TRANZ-feh-race>

Aspartate aminotransferase
<as-PAR-tayt uh-MEE-noh-TRANZ-feh-race>

Bosutinib
<boh-SOO-tih-nib>

Chromosome
<KROH-muh-some>

Chronic myeloid leukemia
<KRAH-nik MY-eh-loyd loo-KEE-mee-uh>

Dasatinib
<da-SA-tih-nib>

Electrolyte
<ee-LEK-troh-lite>

Electrocardiogram
<ee-LEK-troh-KAR-dee-oh-gram>

Imatinib
<ih-MA-tih-nib>

Neutrophil
<NOO-troh-fil>

Nilotinib
<nye-LOH-tih-nib>

Platelet
<PLAYT-let>

Tyrosine kinase inhibitor
<TY-ruh-seen KY-nays in-HIH-bih-ter>

Additional information

More information can be found in the scientific article of this study, which you can access here:

View Scientific Article

Alanine aminotransferase
<A-luh-neen uh-MEE-noh-TRANZ-feh-race>

Aspartate aminotransferase
<as-PAR-tayt uh-MEE-noh-TRANZ-feh-race>

Bosutinib
<boh-SOO-tih-nib>

Chromosome
<KROH-muh-some>

Chronic myeloid leukemia
<KRAH-nik MY-eh-loyd loo-KEE-mee-uh>

Dasatinib
<da-SA-tih-nib>

Electrolyte
<ee-LEK-troh-lite>

Electrocardiogram
<ee-LEK-troh-KAR-dee-oh-gram>

Imatinib
<ih-MA-tih-nib>

Neutrophil
<NOO-troh-fil>

Nilotinib
<nye-LOH-tih-nib>

Platelet
<PLAYT-let>

Tyrosine kinase inhibitor
<TY-ruh-seen KY-nays in-HIH-bih-ter>

Advice for adjusting the amount of bosutinib

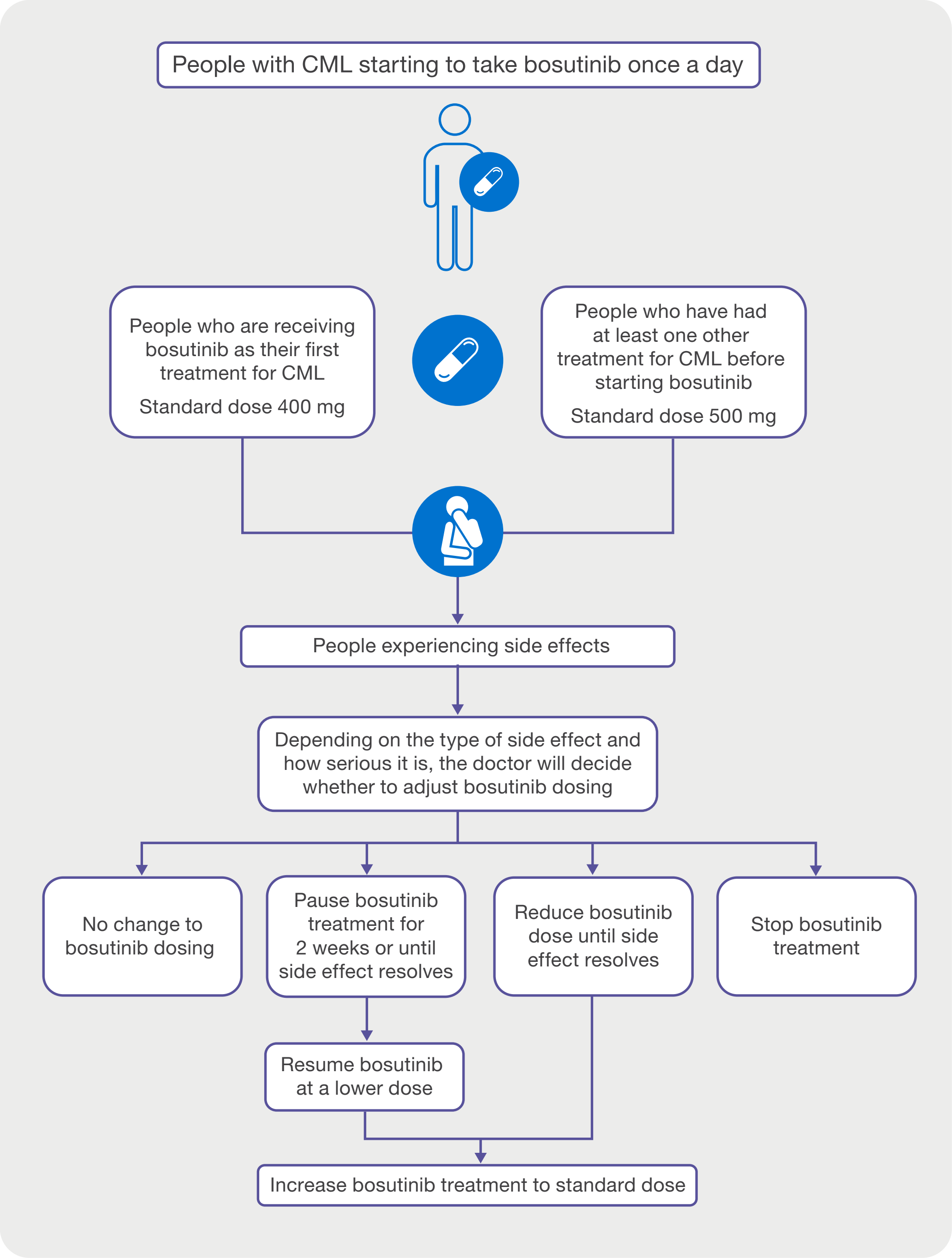

Some people with certain conditions might start bosutinib treatment at a lower amount than the standard recommended dose (for example, at 200 or 300 mg once a day).

To increase the amount of bosutinib after a low starting amount or if the amount has been lowered to manage side effects, your doctor will advise you to increase your dose by 100 mg every 1 to 2 weeks until you are taking the dose that is best for you.

Additional information

More information can be found in the scientific article of this study, which you can access here:

View Scientific Article

More information from this review can be found here: View Scientific Article

Alanine aminotransferase
<A-luh-neen uh-MEE-noh-TRANZ-feh-race>

Aspartate aminotransferase
<as-PAR-tayt uh-MEE-noh-TRANZ-feh-race>

Bosutinib
<boh-SOO-tih-nib>

Chromosome
<KROH-muh-some>

Chronic myeloid leukemia
<KRAH-nik MY-eh-loyd loo-KEE-mee-uh>

Dasatinib
<da-SA-tih-nib>

Electrolyte
<ee-LEK-troh-lite>

Electrocardiogram
<ee-LEK-troh-KAR-dee-oh-gram>

Imatinib
<ih-MA-tih-nib>

Neutrophil
<NOO-troh-fil>

Nilotinib
<nye-LOH-tih-nib>

Platelet
<PLAYT-let>

Tyrosine kinase inhibitor
<TY-ruh-seen KY-nays in-HIH-bih-ter>

What were the main conclusions reported in the review?

- The review provides practical recommendations for how to stop and manage side effects experienced by some people taking bosutinib. The authors of the review are doctors treating people with CML who are taking bosutinib.
- The side effects included in the review affect:
  - Blood cells
  - Heart, veins, and arteries
  - Kidneys
  - Skin
  - Stomach and intestines
  - Liver
  - Lungs.
- There is some doable advice your doctor can give you to stop side effects. They can also advise you to identify the signs and symptoms. This will help you tell your doctor as soon as possible.
- The review has practical advice for doctors:
  - For preventing and treating side effects
  - For lowering or pausing bosutinib dosing to help manage side effects
  - For increasing the dose of bosutinib once a side effect has been treated
  - About when bosutinib should be stopped.
- This review may help doctors treating people with CML taking bosutinib manage their side effects and continue bosutinib treatment.

Who sponsored this review?

Pfizer Inc, 235 East 42nd Street, NY, NY 10017

Phone (United States): +1 212-733-2323

Pfizer thanks all of the people who took part in this study.

Additional information

More information can be found in the scientific article of this study, which you can access here:

View Scientific Article

Further information

For more information on this review article, please visit:

<insert hyperlink to full scientific article on journal website>

For more information on clinical studies in general, please visit:

https://www.clinicaltrials.gov/ct2/about-studies/learn

Writing support for this summary was provided by Daniel East, PhD, Envision Pharma Group, Inc. and was funded by Pfizer. The authors of the review article were involved in preparing this summary.
