# Supplementary material for: Practical considerations in the management of patients treated with bosutinib for chronic myeloid leukemia
Source: Ann Hematol. 2024 Jul 18;103(9):3429–42. doi: 10.1007/s00277-024-05851-4 (PMC11358173; doi:10.1007/s00277-024-05851-4)
Supplement: Supplementary file 2 — Supplementary file2 (DOCX 189 KB) [file 277_2024_5851_MOESM2_ESM.docx]

**Online Resource 1**

**Practical considerations in the management of patients treated with bosutinib for chronic myeloid leukemia**

Jeffrey H Lipton^1^, Tim H Brümmendorf^2,3^, Kendra Sweet^4^, Jane F Apperley^5^, and Jorge E Cortes^6^

*^1^Princess Margaret Cancer Centre, Toronto, Canada; ^2^Department of Hematology, Oncology, Hemostaseology and Stem Cell Transplantation, Faculty of Medicine, RWTH Aachen University Hospital, Aachen, Germany; ^3^Center for Integrated Oncology Aachen Bonn Cologne Düsseldorf (CIO ABCD); ^4^Moffitt Cancer Center, Tampa, FL, USA; ^5^Centre for Haematology, Imperial College London, London, UK; ^6^Division of Hematology and SCT, Georgia Cancer Center, Augusta, GA, USA*

**Supplementary Materials**

**Supplementary Table 1.** AEs of special interest during bosutinib treatment across clinical trials

| **Clinical trial** | **Patient population and bosutinib dose** | **Study** | **Median duration of treatment** | **All grade AEs, %** | **Grade ≥3 AEs, %** | **Median time to onset and duration of AE** | **Discontinuations or dose modifications due to AEs, %** |
| --- | --- | --- | --- | --- | --- | --- | --- |
| ***Hematological events*** | | | | | | | |
| Phase 3 BFORE study  NCT02130557 | Newly diagnosed  CP CML  400 mg QD | Final results, *n*=268 [1]  Long term GI, liver, effusion, renal safety, *n*=268 [2]  Primary results, *n*=268 [3] | 55 months  55 months  14.1 months | Any myelosuppression: 47.8 Thrombocytopenia: 35.8 Anemia: 22.0 Neutropenia: 12.3 Leukopenia: 6.7 Lymphopenia: 5.6  –  Thrombocytopenia: 35.1 Anemia: 18.7 Neutropenia: 11.2 Leukopenia: 5.6 | Thrombocytopenia: 14.2 Anemia: 4.5 Neutropenia: 7.5 Leukopenia: 1.5  –  Thrombocytopenia: 13.8 Anemia: 3.4 Neutropenia: 6.7 Leukopenia: 1.1 | Most TEAEs occurred during the first year of treatment  –  – | Dose reductions: Thrombocytopenia:1.1 Neutropenia: 1.1  Discontinuations: Thrombocytopenia: 1.1 Neutropenia: 1.1  – |
| Phase 3 BELA study  NCT00574873 | Newly diagnosed  CP CML  500 mg QD | Renal safety, *n*=248 [4] | 30.4 months | All myelosuppression AEs: 48.0 Thrombocytopenia: 28.0 Neutropenia: 13.0 Anemia: 25.0 Leukopenia: 9.0 | Thrombocytopenia: 13.0 Neutropenia: 8.0 Anemia: 9.0 Leukopenia: 2.0 | Median time to first event 29 (range: 8–924) days Median duration of event, any grade: 26 (range: 1–1212) days; grade 3/4 to grade 0/1, 22 (range: 2–914) days | All myelosuppression  Discontinuations: 4.0 Dose reductions: 14.0 Dose interruptions: 36.0  Discontinuations due to thrombocytopenia: 2.0 Discontinuations due to neutropenia: 1.0 |
| Phase 4 BYOND study  NCT02228382 | CP CML or AP CML resistant/intolerant to ≥1 TKI  500 mg QD | Preliminary results, n=163 [5] | 23.7 months | Anemia: 15.3 Thrombocytopenia: 11.0 | Anemia: 4.3 Thrombocytopenia: 8.0 | – | – |
| Phase 1/2 Study 200 and extension study    NCT00261846 and NCT01903733 | CP CML resistant/intolerant to imatinib  500 mg QD  CP CML resistant or intolerant to previous TKI therapy or AP/ BP CML, or Ph+ ALL  500 mg QD | Final 10-year results, n=284 [6]  8-year update, n=570 (CP2L, n=284; CP3L, n=119; ADV, n=167); extension study: n=136 (CP2L, n=90; CP3L, n=28; ADV, n=18) [7] | 26 months  CP2L, 26 months CP3L, 9 months ADV, 4 months | Thrombocytopenia: 42.0  Thrombocytopenia: 41 Anemia: 32 Neutropenia: 19 Leukopenia: 12 Anemia newly occurring: Year 10: 3.0 Year 11: 1.0 Year 12: 4.0 | –  – | –  – | Discontinuations: Thrombocytopenia: 6.0 Neutropenia: 2.0  – |
| Phase 1/2 BELA study  NCT00261846 | CP CML resistant or intolerant to previous TKI therapy  500 mg QD | 5-year results 2L CP,  n=284 (IM-R, n=195; IM-I, n=89) [8] | 25.6 months | Thrombocytopenia: 41.5 Anemia: 29.2 Neutropenia: 16.2 Leukopenia: 13.0 | Thrombocytopenia: 25.4 Anemia: 13.4 Neutropenia: 9.9 Leukopenia: 5.3 |  | Discontinuations:  Thrombocytopenia: 6.0 Neutropenia: 2.1 Anemia: 1.1 |
| ***Cardiovascular events*** | | | | | | | |
| Phase 3 BFORE study (NCT02130557) | Newly diagnosed CP CML  400 mg QD | Final results, n=268 [1]  Long term CV safety, n=268 [9]  Primary results, n=268 [3] | 55 months  55 months  14.1 months | Any cardiac: 9.7  Sinus bradycardia: 2.2  ECG QT prolonged: 1.5  Any hypertension: 10.4  Hypertension: 9.7"  Any vascular: 7.5  Cardiovascular: 4.9  Angina pectoris: 3.0  Myocardial ischemia: 2.2  Cerebrovascular: 0.7  Peripheral vascular: 2.2  Cardiac cluster: 9.7  Sinus bradycardia 2.2  Vascular cluster: 7.5  Angina pectoris: 3.0  Hypertension cluster: 10.4  Hypertension: 9.7  Any cardiac event: 5.2  Electrocardiogram QT prolonged: 1.5  Atrial fibrillation: 1.1  Sinus bradycardia: 1.5  Tachycardia: 0.4  Supraventricular tachycardia: 0.4  Bradycardia: 0.7  Ventricular extrasystole: 0.4  Pericardial effusion: 0.4  Extrasystoles: 0.4  Supraventricular extrasystoles: 0.4  Any vascular event: 4.5  Cardiovascular: 3.0  Angina pectoris: 1.5  Myocardial ischemia: 1.5  Acute coronary syndrome: 0.4  Coronary artery disease: 0.4  Coronary artery occlusion: 0.4  Cerebrovascular: 0  Peripheral vascular: 1.5  Angiopathy: 0.4  Capillary fragility: 0.4  Deep vein thrombosis: 0.4  Venous thrombosis limb: 0.4  Hypertension-related event:5.2  Arterial hypertension: 4.9  Hypertensive crisis: 0.4 | Hypertension: 4.5  Grade 5 acute cardiac failure (n=1)  Grade 5 myocardial ischemia (n=1)  Any cardiac event: 1.1  Electrocardiogram QT prolonged: 0.4  Atrial fibrillation: 0.4  Supraventricular tachycardia: 0.4  Pericardial effusion: 0.4  Any vascular event: 1.5  Cardiovascular: 1.5  Angina pectoris: 0.4  Acute coronary syndrome: 0.4  Coronary artery disease: 0.4  Coronary artery occlusion: 0.4  Hypertension-related event: 1.9  Arterial hypertension: 1.9 | Most TEAEs occurred during the first year of treatment  –  – | Discontinuations:  Cardiac: 1 (0.4%)  Vascular: 3 (1.1%)  Discontinuations:  Cardiac cluster: 0.4  Vascular cluster: 1.1  Hypertension cluster: 0  – |
| Phase 3 BELA study  NCT00574873 | Newly diagnosed CP CML  500 mg QD | Renal safety, n=248 [4] | 30.4 months | Any cardiovascular: 10.0  Any cardiac: 8.0  Palpitations: 2.0  Pericardial effusion: 2.0  Any vascular: 10.0  Hypertension: 6.0  Hematoma: 2.0 | – | Median time to first event 166 (range: 1–1023) days Median duration of event, 14 (range: 1–750) days | Discontinuations: 2.0 Dose reductions: 15.0 Dose interruptions: 31.0 |
| Phase 4 BYOND study  NCT02228382 | CP CML or AP CML resistant/intolerant to ≥1 TKI  500 mg QD | Primary results, n=163 [5] | 23.7 months | Any cardiac TEAE: 14.7 Cardiac disorders: 14.1 Cardiac failure: 3.7 Atrial fibrillation: 3.1 Tachycardia: 1.8 Arrhythmia: 1.2 Bradycardia: 1.2 Cardiac failure congestive: 1.2 Atrial flutter: 0.6 Atrioventricular block complete: 0.6 Bundle branch block right: 0.6 Cardiac failure acute: 0.6 Cardiac flutter: 0.6 Cardiogenic shock: 0.6 Extrasystoles: 0.6 Sinus bradycardia: 0.6 Investigations: 0.6 Electrocardiogram QT interval prolonged: 0.6  Any vascular TEAE: 11.7 Cardiovascular: 3.1 Angina pectoris: 1.2 Angina unstable: 0.6 Coronary artery occlusion: 0.6 Myocardial ischemia: 0.6 Cerebrovascular: 3.1 Cerebrovascular accident: 1.2 Transient ischemic attack: 1.2 Carotid artery stenosis: 0.6 Peripheral vascular: 6.1 Peripheral arterial occlusive disease: 1.8 Peripheral ischemia: 1.2 Aortic stenosis: 0.6 Arterial rupture: 0.6 Intermittent claudication: 0.6 Peripheral coldness: 0.6 Vascular pain: 0.6 | – | – | – |
| Phase 1/2 Study 200 and extension study    NCT00261846 and NCT01903733 | CP CML resistant/intolerant to imatinib  500 mg QD  CP CML resistant or intolerant to previous TKI therapy or AP/BP CML, or ALL  500 mg QD  CP CML resistant or intolerant to previous TKI therapy or AP/ BP CML, or Ph+ ALL  500 mg QD | Final 10-year results, n=284 [6]  Long-term cardiac, vascular, hypertension, and effusion, n=570 (CP2L, n=284; CP3L, n=119; ADV, n=167) [10]  8-year update, n=570 (CP2L, n=284; CP3L, n=119; ADV, n=167); extension study: n=136 (CP2L, n=90; CP3L, n=28; ADV, n=18) [7] | 26 months  11.1 months  CP2L, 26 months CP3L, 9 months ADV, 4 months | Cardiac: 12.0  Vascular: 11.0  Cardiac cluster: 10.9 Atrial fibrillation: 3.0 Cardiac failure congestive: 2.6 Tachycardia: 1.8 Cardiac failure: 1.6 Bradycardia: 1.2 Newly occurring Year 5: 3.6 Year 6: 2.6 Year 7: 2.2 Year 8: 0  Vascular cluster: 8.8 Cardiovascular: 4.4 Angina pectoris: 1.6 Coronary artery disease: 1.4 Cerebrovascular: 3.2 Peripheral vascular: 2.3 Newly occurring Year 5: cardiovascular, 1.8; cerebrovascular, 0.6; peripheral vascular, 1.2 Year 6: cardiovascular, 1.3; cerebrovascular, 0.7; peripheral vascular, 0  Hypertension cluster: 9.1 Hypertension: 8.2 Newly occurring Year 5: 1.2 Year 6: 0 Year 7: 1.5 Year 8: 0  Hypertension newly occurring: Year 10: 1.0 Year 11: 3.0 Year 12: 2.0 | –  Cardiac cluster: 4.6 Vascular cluster: 5.8 Hypertension cluster: 3.  – | –  Median time to first event Cardiac cluster: 92 (range:1–2885) days  Vascular cluster: 400 (range: 8–3208) days  Hypertension cluster: 610 (range: 1–3404) days  – | –  Cardiac cluster:  Discontinuations: 0.9  Dose interruption: 21.0 Dose reductions: 6.5  Vascular cluster:  Discontinuations: 1.2  Dose interruption: 16.0 Dose reductions: 6.0  Hypertension cluster:  Discontinuations: 0  Dose interruption: 7.7 Dose reductions: 0  – |
| Phase 1/2 BELA study  NCT00261846 | CP CML resistant or intolerant to previous TKI therapy  500 mg QD | 5-year results 2L CP:  CP2L n=284 (IM-R, n=195; IM-I, n=89) [8] | 25.6 months | Cardiac AEs:13.0 Pericardial effusion: 3.0 Congestive cardiac failure: 2.0 Atrial fibrillation: 2.0 Bradycardia: 2.0 Cardiac failure: 2.0  Vascular AEs: 7.7  Hypertension-related AEs: 9.2 | Cardiac AEs: 5.6 Pericardial effusion: 1.0 Congestive cardiac failure: 2.0 Atrial fibrillation: 1.0 Bradycardia: 1.0 Cardiac failure: 1.0  Vascular AEs: 3.9  Hypertension-related AEs: 2.8 | Cardiac: median time to first occurrence: 184 (range: 1–2563) days  Vascular: median time to first occurrence: 548 (range: 47–2452) days | Discontinuations:  Cardiac failure: 0.4  Coronary artery disease: 0.4 |
| ***Renal dysfunction*** | | | | | | | |
| Phase 3 BFORE study  (NCT02130557) | Newly diagnosed CP CML  400 mg QD | Final results, n=268 [1]  Long term GI, liver, effusion, renal safety, n=268 [2, 11] | 55 months  55 months | Any renal: 10.4 Blood creatinine increased: 6.7 Acute kidney injury: 2.2  Renal cluster: 10.4 Blood creatinine increased: 6.7 Acute kidney injury: 2.2 | –  Renal cluster: 2.2 Blood creatinine increased: 0.4 Acute kidney injury: 1.1 | Most TEAEs occurred during the first year of treatment  Increased incidence in some TEAEs in later years | –  Renal cluster: Discontinuations: 11.1 Dose reduction: 17.9 Dose interruption: 32.1 |
| Phase 1/2 BELA study  (NCT00261846) | CP CML resistant or intolerant to previous TKI therapy or AP CML, or BP CML, or ALL  500 mg QD | Renal safety, n=570 (CP, n=403; ADV, n=167) [12]  5-year results 2L CP, n=284 (IM-R, n=195; IM-I, n=89) [8] | CP, 18.1 months  ADV, 3.95 months  25.6 months | CP All renal AEs: 13.0 Increased blood creatinine: 10.0 Abnormal blood creatinine: <1.0 Renal impairment: 1.0 Renal failure: 2.0 Acute renal failure: 1.0 Chronic renal failure: <1.0 Acute prerenal failure: <1.0 Anuria: <1.0 Oliguria: <1.0  ADV All renal AEs: 13.0 Increased blood creatinine: 5.0 Renal impairment: 1.0 Renal failure: 5.0 Acute renal failure: 2.0 Chronic renal failure: 1.0 Acute prerenal failure: 1.0  Renal AEs: 13.0 Blood creatinine increased:  Newly occurring in year 3: 5.0  Newly occurring in year 4: 5.0 | CP All renal AEs: 2.0 Increased blood creatinine: <1.0 Renal impairment: <1.0 Renal failure: 1.0 Acute renal failure: 1.0 Acute prerenal failure: <1.0  ADV All renal AEs: 4.0 Increased blood creatinine: 1.0 Renal failure: 1.0 Acute renal failure: 2.0 Chronic renal failure: 1.0 Acute prerenal failure: 1.0  Renal AEs: 2.1 | CP Median time to first event: 497 (range: 1–2695) days  Median cumulative duration of events: 128 (range: 1–1167) days  ADV Median time to first event: 29 (range: 1–1176) days  Median cumulative duration of events: 38 (range: 5–1406) day  Median time to first occurrence: 673 (range: 8–2695) days | CP Discontinuations: 1.0 Dose reductions: 4.0  Dose interruption: 13.0  ADV Discontinuations: <1.0 Dose reductions: 5.0 Dose interruption: 19.0  Discontinuations: Renal failure: 0.4 Increased blood creatinine: 0.4 |
| Phase 1/2 Study 200 and extension study  NCT00261846 and NCT01903733 | CP CML resistant or intolerant to previous TKI therapy or AP/ BP CML, or ALL  500 mg QD | 8-year update, n=570 (CP2L, n=284; CP3L, n=119; ADV, n=167); extension study: n=136 (CP2L, n=90; CP3L, n=28; ADV, n=18) [7] | CP2L, 26 months CP3L, 9 months ADV, 4 months | Blood creatinine increased: 11 Newly occurring: Year 10: 0 Year 11: 3.0 Year 12: 0 | – | – | – |
| Phase 3 BELA study renal safety  NCT00574873 | Newly diagnosed CP CML  500 mg QD | Renal safety, n=248 [12] | 54.4 months | All renal AEs: 9.0 Increased blood creatinine: 6.0 Renal impairment: <1.0  Renal failure: 1.0 Acute renal failure: 2.0 Chronic renal failure: 1.0 Oliguria: 1.0 | All renal AEs: 2.0 Increased blood creatinine: <1.0 Renal failure: <1.0 Acute renal failure: 1.0 Chronic renal failure: <1.0 | All renal Median (range) time to first event 421 (7–1765) days  Median (range) cumulative duration of events, 25 (2–923) days | All renal Discontinuations: 0 Dose reductions: 9.0 Dose interruptions: 23.0 |
| Phase 4 BYOND study  NCT02228382 | CP CML or AP CML resistant/intolerant to ≥1 TKI | Primary results, n=163 [5] | 23.7 months | Blood creatinine increased: 14.7 | – | – | – |
| ***Skin toxicities*** | | | | | | | |
| Phase 3 BFORE study  NCT02130557 | Newly diagnosed CP CML  400 mg QD | Final results, n=268 [1]  Long term GI, liver, effusion, renal safety, n=268 [2]  Primary results, n=268 [3] | 55 months  55 months  14.1 months | Any rash: 39.2 Rash: 23.1 Rash maculo-papular: 5.2 Erythema: 4.9 Rash pruritic: 3.7 Dermatitis acneiform: 3.4 Acne: 3.0 Eczema: 2.6  –  Rash: 19.8 | Rash: 0.7  –  Rash: 0.4 | Most TEAEs occurred during the first year of treatment  –  – | –  Discontinuations: Rash: 0.4  – |
| Phase 3 BELA study  NCT00574873 | Newly diagnosed CP CML  500 mg QD | Renal safety, n=248 [4] | 30.4 months | Rash/skin toxicity: 35.0 Rash: 25.0 | Rash: 2.0 | Median time to first event: 57.5 (range: 1–931) days  Median duration of event: 22 (range: 1–552) days | Discontinuations: 1 Dose reductions: 13 Dose interruptions: 22 |
| Phase 4 BYOND study  (NCT02228382) | CP CML or AP CML resistant/intolerant to ≥1 TKI  500 mg QD | Primary results, n=163 [5] | 23.7 months | Rash: 15.3 | Rash: 4.3 | – | – |
| Phase 1/2 Study 200 and extension study    (NCT00261846 and NCT01903733) | CP CML resistant or intolerant to previous TKI therapy or AP/BP CML, or ALL  500 mg QD | 8-year update, n=570 (CP2L, n=284; CP3L, n=119; ADV, n=167); extension study: n=136 (CP2L, n=90; CP3L, n=28; ADV, n=18) [7] | CP2L, 26 months CP3L, 9 months ADV, 4 months | Rash: 34.0 | – | – | – |
| Phase 1/2 BELA study  NCT00261846 | CP CML resistant or intolerant to previous TKI therapy  500 mg QD | 5-year results 2L CP, n=284 (IM-R, n=195; IM-I, n=89) [8] | 25.6 months | Rash: 36.3 | Rash: 9.2 | – | Discontinuations:  Rash: 1.1 |
| ***Diarrhea*** | | | |  | | | |
| Phase 3 BFORE study  (NCT02130557) | Newly diagnosed CP CML  400 mg QD | Final results, n=268 [1]  Long term GI, liver, effusion, renal safety, n=268, [2, 11]  Primary results, n=268 [3] | 55 months  55 months  14.1 months | Diarrhea: 75.0  Diarrhea: 75.0  Diarrhea: 70.1 | Diarrhea: 9.0  Diarrhea: 9.0  Diarrhea: 7.8 | Most TEAEs occurred during the first year of treatment  Onset occurred primarily in year 1  Median cumulative duration: 15 (range: 1 to 447) days  Median time to first event: 3 (range: 1 to 505) days | –  Discontinuations:  Diarrhea: 1.5  Dose interruptions and reductions: 13.3  Discontinuations: 0.7 |
| Phase 3 BELA study  NCT00574873 | Newly diagnosed CP CML  500 mg QD | Renal safety, n=248 [4] | 30.4 months | Diarrhea: 70.0 | Diarrhea: 12.0 | Median time to first event: 3 (range: 1–591) days  Median duration of event, any grade, 3 (range: 1–836) days; grade 3/4 to grade 0/1, 8 (range: 2–103) days | Discontinuations: 0  Dose reductions: 8.0  Dose interruptions: 21.0 |
| Phase 4 BYOND study  NCT02228382 | CP CML or AP CML resistant/intolerant to ≥1 TKI  500 mg QD | Primary results, n=163 [5] | 23.7 months | Diarrhea: 87.7 | Diarrhea: 16.0 | Median time to first TEAE of diarrhea: 2.0 (1–304) days  Median duration of diarrhea event (any grade): 8.0 (range: 1–715) days | Discontinuations: 1.2 |
| Phase 1/2 Study 200 and extension study    NCT00261846 and NCT01903733 | CP CML resistant/intolerant to imatinib  500 mg QD  CP CML resistant or intolerant to previous TKI therapy or AP/ BP CML, or ALL  500 mg QD | Final 10-year results, n=284 [6]  8-year update, n=570 (CP2L, n=284; CP3L, n=119; ADV, n=167); extension study: n=136 (CP2L, n=90; CP3L, n=28; ADV, n=18) [7] | 26 months  CP2L, 26 months CP3L, 9 months ADV, 4 months | Diarrhea: 86.0  Diarrhea: 82.0 | –  – | –  – | –  – |
| Phase 1/2 BELA study  NCT00261846 | CP CML resistant or intolerant to previous TKI therapy  500 mg QD | 5-year results 2L CP, n=284 (IM-R, n=195;  IM-I, n=89) [8] | 25.6 months | Diarrhea: 85.6 | Diarrhea: 9.5 | Most diarrhea AEs occurred in year 1, only 4 patients experienced diarrhea for the first time in years 2–5  Median time to first occurrence: 2 (range:  1–1330) days | Discontinuations: Diarrhea: 1.4 |
| ***Other GI toxicities*** | | | | | | | |
| Phase 3 BFORE study  NCT02130557 | Newly diagnosed CP CML  400 mg QD | Final results, n=268 [1]    Long term GI, liver, effusion, renal safety, n=268 [2, 11]  Primary results, n=268 [3] | 55 months  55 months  14.1 months | Nausea: 37.3  Abdominal pain: 22.8  Vomiting: 20.5  Constipation: 13.4  Abdominal pain upper: 10.4  GI cluster (includes diarrhea): 79.9  Nausea: 37.3  Vomiting: 20.5    Nausea: 35.1  Vomiting: 17.9  Abdominal pain: 17.9 | Abdominal pain: 1.9  Vomiting: 1.1  GI cluster (includes diarrhea): 9.0  Nausea: 0  Vomiting: 1.1  Vomiting: 1.1  Abdominal pain: 1.9 | Most TEAEs occurred during the first year of treatment  Onset occurred primarily in year 1  – | Discontinuations:  Nausea: 0  Vomiting: 0  GI cluster (includes diarrhea): Discontinuations: 9.1  Dose reduction: 11.2  Dose interruption: 20.6  – |
| Phase 3 BELA study  NCT00574873 | Newly diagnosed CP CML  500 mg QD | Renal safety, n=248 [4] | 30.4 months | Any GI (including diarrhea): 76.0  Vomiting: 33.0  Nausea: 32.0  Upper abdominal pain: 15.0  Abdominal pain: 14.0 | Vomiting: 3.0 Nausea: 1.0 Abdominal pain: 1.0 | Most events were transient | Discontinuations:  Vomiting: 2.0 |
| Phase 4 BYOND study  NCT02228382 | CP CML or AP CML resistant/intolerant to ≥1 TKI  500 mg QD | Primary results, n=163 [5] | 23.7 months | Any GI: 91.4  Abdominal pain: 41.1  Nausea: 39.9  Vomiting: 32.5  Constipation: 17.2  Abdominal pain upper: 22.1 | Abdominal pain: 4.3  Nausea: 2.5  Vomiting: 3.7  Abdominal pain upper: 1.2 | – | Discontinuations:  Nausea: 1.8  Vomiting: 1.2 |
| Phase 1/2 Study 200 and extension study    NCT00261846 and NCT01903733 | CP CML resistant/intolerant to imatinib  500 mg QD  CP CML resistant or intolerant to previous TKI therapy or AP/ BP CML, or ALL  500 mg QD | Final 10-year results, n=284 [6]  8-year update, n=570 (CP2L, n=284; CP3L, n=119; ADV, n=167); extension study: n=136 (CP2L, n=90; CP3L, n=28; ADV, n=18) [7] | 26 months  CP2L, 26 months CP3L, 9 months ADV, 4 months | Nausea: 46.0  Nausea: 47  Vomiting: 40  Abdominal pain: 25  Abdominal pain upper: 17  Constipation: 15 | –  – | –  – | –  – |
| Phase 1/2 BELA study  NCT00261846 | CP CML resistant or intolerant to previous TKI therapy  500 mg QD | 5-year results 2L CP, n=284 (IM-R, n=195; IM-I, n=89) [8] | 25.6 months | Nausea: 46.1 Vomiting: 37.3  Abdominal pain: 27.1  Upper abdominal pain: 20.8  Constipation: 13.7 | Nausea: 1.8  Vomiting: 3.9  Abdominal pain: 2.1  Upper abdominal pain: 0.4  Constipation: 0.4 | – | – |
| ***Liver enzyme elevations*** | | | | | | | |
| Phase 3 BFORE study  NCT02130557 | Newly diagnosed CP CML  400 mg QD | Final results, n=268 [1]  Long term GI, liver, effusion, renal safety, n=268 [2, 11]  Primary results, n=268 [3] | 55 months  55 months  14.1 months | Any liver TEAE: 44.0 ALT increased: 33.6 AST increased: 25.7 Blood bilirubin increased: 6.3 Blood alkaline phosphatase increased: 6.3 Transaminases increased: 3.0 Hyperbilirubinemia: 2.2  Liver cluster: 44.0 Increased ALT: 33.6 Increased AST: 25.7 Blood bilirubin increased: 6.3 Blood alkaline phosphate increased: 6.3 Transaminases increased: 3.0  Hyperbilirubinemia: 2.2 (Most common TEAE newly occurring after 1 year was increased lipase: 9.0)  Any liver AE: 39.9 Increased ALT: 30.6 Increased AST: 22.8 | ALT increased: 20.9 AST increased: 10.4 Lipase increased: 13.4 Blood creatine phosphokinase increased: 1.9  Liver cluster: 26.9 Increased ALT: 20.9 Increased AST: 10.4 Blood bilirubin increased: 1.1 Transaminases increased: 1.1  Any liver AE: 24.3 Increased ALT: 19.0 Increased AST: 9.7 | Most TEAEs occurred during the first year of treatment  Onset occurred primarily in year 1  Median time to first event: 30 (range: 8 to 421) days  Median duration of event: 18 (range: 1 to 442) days | Discontinuations:  ALT increased: 13 (4.9%) (year 1 4.5%) AST increased: 7 (2.6%) Lipase increased: 5 (1.9%) (year 1, 0.7)  Discontinuations:  Liver cluster: 8.5 Increased ALT: 4.9 (4.5 at year 1) Increased AST: 2.6 Lipase increased: 1.9 Hepatotoxicity: 0.7  Discontinuations after year 1:  Increased lipase: 1.9 (0.7 year 1)  Dose reduction: 26.3 Dose interruption: 60.2  Discontinuations: Increased ALT: 4.9 Increased AST: 2.2  Dose interruptions: 25.7 Dose reductions: 8.6 |
| Phase 3 BELA study renal safety  NCT00574873 | Newly diagnosed CP CML  500 mg QD | Renal safety, n=248 [4] | 30.4 months | Increased ALT: 33.0 Increased AST: 28.0 Increased lipase: 15.0 | Increased ALT: 19.0 Increased AST: 8.0 Increased lipase: 9.0 | Increased ALT Median time to first event 28 (range: 7–1091) days Median duration of event, any grade, 17 (range: 1–421) days; grade 3/4 to grade 0/1, 21.5 (range: 5–207) days  Increased AST Median time to first event 29 (range: 7–1091) days Median duration of an event, any grade, 14 (range: 1–496) days; grade 3/4 to grade 0/1, 21 (range: 3–56) days | Increased ALT Discontinuations: 4.0 Dose reductions: 31.0 Dose interruptions: 57.0  Increased AST Discontinuations: 0 Dose reductions: 13.0 Dose interruptions: 41.0  Increased lipase: Discontinuations: 1.0 |
| Phase 4 BYOND study  NCT02228382 | CP CML or AP CML resistant/intolerant to ≥1 TKI  500 mg QD | Primary results, n=163 [5] | 23.7 months | Increased ALT: 25.8 Increased AST: 19.6 Increased lipase: 14.1 | Increased ALT: 14.1 Increased AST: 4.3 Increased lipase: 6.7 | – | Discontinuations: Increased ALT: 4.9 Increased AST: 2.5 |
| Phase 1/2 Study 200 and extension study  NCT00261846 and NCT01903733 | CP CML resistant/intolerant to imatinib  500 mg QD  CP CML resistant or intolerant to previous TKI therapy or AP/ BP CML, or Ph+ ALL  500 mg QD | Final 10-year results, n=284 [6]  8-year update, n=570 (CP2L, n=284; CP3L, n=119; ADV, n=167); extension study: n=136 (CP2L, n=90; CP3L, n=28; ADV, n=18) [7] | 26 months  CP2L, 26 months CP3L, 9 months ADV, 4 months | –  ALT increased: 18 AST increased: 15 | –  – | –  – | Discontinuations:  Increased ALT: 2.0  – |
| Phase 1/2 BELA study  NCT00261846 | CP CML resistant or intolerant to previous TKI therapy  500 mg QD | 5-year results 2L CP, n=284 (IM-R, n=195;  IM-I, n=89) [8] | 25.6 months | ALT increased: 22.2 AST increased: 19.7 | ALT increased: 8.5 AST increased: 3.9 | – | Discontinuations:  ALT increased: 2.1 AST increased: 1.1 Lipase increased: 0.4 |
| ***Pulmonary toxicities*** | | | | | | | |
| Phase 3 BFORE study  NCT02130557 | Newly diagnosed CP CML  400 mg QD | Final results, n=268 [1]  Long term GI, liver, effusion, renal safety, n=268 [2, 11]  Primary results, n=268 [3] | 55 months  55 months  14.1 months | Pleural effusion: 5.2 Cough: 11.2 Dyspnea: 10.8  Pleural effusion: 5.2  Pleural effusion:1.9 | Dyspnea: 0.7  Pleural effusion: 0.7  – | Most TEAEs occurred during the first year of treatment  –  – | –  Discontinuations: Pleural effusion: 0.7 Pneumonia: 0.7  – |
| Phase 3 BELA study  NCT00574873 | Newly diagnosed CP CML  500 mg QD | renal safety, n=248 [4] | 30.4 months | Pleural effusion: 4.0 | – | – | Discontinuations:  Pleural effusion: 1.0 |
| Phase 4 BYOND study  NCT02228382 | CP CML or AP CML resistant/intolerant to ≥1 TKI  500 mg QD | Primary results, n=163 [5] | 23.7 months | Pleural effusion: 16.6 | Pleural effusion: 6.1 | – | – |
| Phase 1/2 Study 200 and extension study  NCT00261846 and NCT01903733 | CP CML resistant/intolerant to imatinib  500 mg QD  CP CML resistant or intolerant to previous TKI therapy or AP/ BP CML, or ALL  500 mg QD  CP CML resistant or intolerant to previous TKI therapy or AP/ BP CML, or ALL  500 mg QD | Final 10-year results, n=284 [6]  Long-term cardiac, vascular, hypertension, and effusion, n=570 (CP2L, n=284; CP3L, n=119; ADV, n=167) [10]  8-year update, n=570 (CP2L, n=284; CP3L, n=119; ADV, n=167); extension study: n=136 (CP2L, n=90; CP3L, n=28; ADV, n=18) [7] | 26 months  11.1 months  CP2L, 26 months; CP3L, 9 months; ADV, 4 months | Pleural effusion: 13.0  Pleural effusion: 11.9  Pleural effusion: 13.0 Newly occurring: Year 10: 1.0 Year 11: 3.0 Year 12: 6.0 | –  –  – | –  –  – | –  –  – |
| Phase 1/2 BELA study  (NCT00261846) | CP CML resistant or intolerant to previous TKI therapy  500 mg QD: | 5-year results 2L CP, n=284 (IM-R, n=195; IM-I, n=89) [8] | 25.6 months | Pleural effusion: 10.6 Pleural effusion:  Newly occurring in year 4: 5.0 | Pleural effusion: 3.2 | – | Discontinuations: Pulmonary hypertension: 0.4 |
| Abbreviations: 1L, first line; 2L second line; 3L, third line; 4L, fourth line; ADV, advanced; AE, adverse event; ALL, acute lymphoblastic leukemia; AP, accelerated phase; AST, aspartate aminotransferase; BP, blast phase; CML, chronic myeloid leukemia; CP, chronic phase; IM-I; imatinib intolerant; IM-R, imatinib resistant; Ph+, Philadelphia chromosome–positive; QD, once daily; TKI, tyrosine kinase inhibitor | | | | | | | |

.

**Supplementary Table 2.** AEs of special interest during bosutinib treatment across real-world studies

| **Study (reference)** | **Patient population** | **Bosutinib dose** | **Duration** | **All grade AEs, %** | **Grade ≥3 AEs, %** | **Median time to onset and duration of AE** | **Discontinuations or dose modifications due to AEs** |
| --- | --- | --- | --- | --- | --- | --- | --- |
| ***Hematological events*** | | | | | | | |
| Real-world study in UK and Netherlands [13] | Ph+ CML | 100–500 mg/day; n=87  Median dose intensity: 324.5 (range: 31.8–568.3) | 15.6 months | Thrombocytopenia: 8.0 Anemia: 3.0 | Thrombocytopenia: 5.0  Anemia: 1.0 |  | Thrombocytopenia N=5 discontinuations or dose reductions N=2 dose reductions due to grade 3/4 AE  Neutropenia N=2 discontinuations or dose reductions N=1 dose reductions due to grade 3/4 AE |
| Retrospective observational analysis in Spain and UK [14] | Ph+ 4L CP, BP, or AP CML | 500 mg/day; n=62  Median dose intensity: 450 (range: 150–550) mg/day | 9.1 months | Anemia: 21.0  Thrombocytopenia: 21.0  Neutropenia: 9.7 | Anemia (grade 2–4): 3.0  Thrombocytopenia (grade 2–4): 6.0  Neutropenia (grade 2–4): 6.0 |  |  |
| ***Cardiovascular events*** | | | | | | | |
| Real-world study in UK and Netherlands [13] | Ph+ CML | 100–500 mg/day; n=87  Median dose intensity: 324.5 (range: 31.8–568.3) | 15.6 months | Chest pain: 9.0  Atrial fibrillation: 3.0  Cardiac failure: 3.0 | Atrial fibrillation: 1.0  Cardiac failure: 1.0 |  | Cardiac failure N=3 discontinuations or dose reductions  N=1 dose reduction due to grade 3/4 AE  Chest pain  N=5 discontinuations or dose reductions  Hypertension N=1 discontinuations or dose reductions |
| Retrospective observational analysis in Spain and UK [14] | Ph+ 4L CP, BP, or AP CML | 500 mg/day; n=62  Median dose intensity: 450 (range: 150–550) mg/day | 9.1 months | Cardiovascular events: 4.8 | Cerebrovascular disease (grade 2–4): 3.2  Occlusive peripheral artery disease (grade 2–4): 1.6  Hypertension (grade 2–4): 3.2 |  |  |
| ***Renal dysfunction*** | | | | | | | |
| Real-world study in UK and Netherlands [13] | Ph+ CML | 100–500 mg/day; n=87  Median dose intensity: 324.5 (range: 31.8–568.3) | 15.6 months | Blood creatinine increased: 6.0 |  |  | Creatinine increased  N=3 discontinuations or dose reductions |
| ***Skin toxicities*** | | | | | | | |
| Real-world study in UK and Netherlands [13] | Ph+ CML | 100–500 mg/day; n=87  Median dose intensity: 324.5 (range: 31.8–568.3) | 15.6 months | Rash: 17.0 |  |  | Rash: N=6 discontinuations or dose reductions  Acne: N=1 discontinuations or dose reductions |
| Retrospective observational analysis in Spain and UK [14] | Ph+ 4L CP, BP, or AP CML | 500 mg/day; n=62  Median dose intensity: 450 (range: 150–550) mg/day | 9.1 months | Rash: 8.1 | Rash (grade 2–4): 8.1 |  |  |
| ***Diarrhea*** | | | | | | | |
| Real-world study in UK and Netherlands [13] | Ph+ CML | 100–500 mg/day; n=87  Median dose intensity: 324.5 (range: 31.8–568.3) | 15.6 months | Diarrhea: 52.0 | Diarrhea: 3.0 |  | N=16 discontinuations or dose reductions  N=1 temporary discontinuation due to grade 3/4 AE  N=1 dose reduction due to grade 3/4 AE |
| Real-world study in UK and Netherlands [15] | CML | Most common starting dose was 300 mg/day, n=53 | 15.6 months | Diarrhea: 54.7 | Diarrhea: 3.8 |  |  |
| Retrospective observational analysis in Spain and UK [14] | Ph+ 4L CP, BP, or AP CML | 500 mg/day; n=62  Median dose intensity: 450 (range: 150–550) mg/day | 9.1 months | Diarrhea: 38.7 | Diarrhea (grade 2–4): 38.7 |  |  |
| ***Other GI toxicities*** | | | | | | | |
| Real-world study in UK and Netherlands [13] | Ph+ CML | 100–500 mg/day; n=87  Median dose intensity: 324.5 (range: 31.8–568.3) | 15.6 months | Nausea: 21.0 Vomiting: 6.0  Abdominal pain: 9.0 Dyspepsia: 5.0 | Vomiting: 1.0 |  | Nausea N=9 discontinuations or dose reductions  Vomiting N=3 discontinuations or dose reductions  Abdominal pain N=2 discontinuations or dose reductions  Abdominal distension N=1 discontinuations or dose reductions |
| Retrospective observational analysis in Spain and UK [14] | Ph+ 4L CP, BP, or AP CML | 500 mg/day; n=62  Median dose intensity: 450 (range: 150–550) mg/day | 9.1 months |  | Abdominal pain (grade 2–4): 9.7 |  |  |
| ***Liver enzyme elevations*** | | | | | | | |
| Real-world study in UK and Netherlands [13] | Ph+ CML | 100–500 mg/day; n=87  Median dose intensity: 324.5 (range: 31.8–568.3) | 15.6 months | Increased ALT: 17.0 | Increased ALT: 3.0 |  | Increased ALT N=23 discontinuations or dose reductions N=1 temporary discontinuation due to grade 3/4 AE  N=1 dose reduction due to grade 3/4 AE  Hepatotoxicity N=2 discontinuations or dose reductions N=1 dose reduction due to grade 3/4 AE |
| Retrospective observational analysis in Spain and UK [14] | Ph+ 4L CP, BP, or AP CML | 500 mg/day; n=62  Median dose intensity: 450 (range: 150–550) mg/day | 9.1 months | Elevated liver enzymes: 12.9 | Elevated liver enzymes (grade 2–4): 12.9 |  |  |
| ***Pulmonary toxicities*** | | | | | | | |
| Real-world study in UK and Netherlands [13] | Ph+ CML | 100–500 mg/day; n=87  Median dose intensity: 324.5 (range: 31.8–568.3) | 15.6 months | Pleural effusion: 5.0 |  |  | N=8 discontinuations or dose reductions |
| Retrospective observational analysis in Spain and UK [14] | Ph+ 4L CP, BP, or AP CML | 500 mg/day; n=62  Median dose intensity: 450 (range: 150–550) mg/day | 9.2 months | Pleural effusion: 11.3 | Pleural effusion (grade 2–4): 11.3 |  |  |

Abbreviations: 1L, first line; 2L second line; 3L, third line; 4L, fourth line; AE, adverse event; AP, accelerated phase; AST, aspartate aminotransferase; BP, blast phase; CML, chronic myeloid leukemia; CP, chronic phase; Ph+, Philadelphia chromosome–positive; UK, United Kingdom.

**References**

1. Brümmendorf TH, Cortes JE, Milojkovic D et al (2022) Bosutinib versus imatinib for newly diagnosed chronic phase chronic myeloid leukemia: final results from the BFORE trial. Leukemia 36:1825-1833. <https://doi.org/10.1038/s41375-022-01589-y>

2. Cortes JE, Milojkovic D, Gambacorti-Passerini C et al (2022) Bosutinib in newly diagnosed chronic myeloid leukemia: gastrointestinal, liver, effusion and renal safety characterization in the BFORE trial (P717). HemaSphere 6:612-613. <https://doi.org/10.1097/01.Hs9.0000845752.32594.93>

3. Cortes JE, Gambacorti-Passerini C, Deininger MW et al (2018) Bosutinib Versus Imatinib for Newly Diagnosed Chronic Myeloid Leukemia: Results From the Randomized BFORE Trial. J Clin Oncol 36:231-237. <https://doi.org/10.1200/JCO.2017.74.7162>

4. Gambacorti-Passerini C, Cortes JE, Lipton JH et al (2014) Safety of bosutinib versus imatinib in the phase 3 BELA trial in newly diagnosed chronic phase chronic myeloid leukemia. Am J Hematol 89:947-953. <https://doi.org/10.1002/ajh.23788>

5. Hochhaus A, Gambacorti-Passerini C, Abboud C et al (2020) Bosutinib for pretreated patients with chronic phase chronic myeloid leukemia: primary results of the phase 4 BYOND study. Leukemia 34:2125-2137. <https://doi.org/10.1038/s41375-020-0915-9>

6. Gambacorti-Passerini C (2021) Second-line bosutinib (BOS) for patients (pts) with chronic phase (CP) chronic myeloid leukemia (CML): Final 10-year results of a phase 1/2 study. J Clin Oncol 39:Abstract-7009.

7. Brümmendorf TH, Cortes JE, Goh YT et al (2020) Bosutinib (BOS) for chronic phase (CP) chronic myeloid leukemia (CML) after imatinib (IMA) failure: ≥8-y update of a phase I/II study. JCO 38(Suppl 15):7549. <https://doi.org/10.1200/JCO.2020.38.15_suppl.7549>

8. Gambacorti-Passerini C, Cortes JE, Lipton JH et al (2018) Safety and efficacy of second-line bosutinib for chronic phase chronic myeloid leukemia over a five-year period: final results of a phase I/II study. Haematologica 103:1298-1307. <https://doi.org/10.3324/haematol.2017.171249>

9. Cortes JE, le Coutre PD, Gambacorti-Passerini C et al (2020) Long-Term Cardiac, Vascular, and Hypertension Safety of Bosutinib (BOS) Versus Imatinib (IMA) for Newly Diagnosed Chronic Myeloid Leukemia (CML): Results from the Bfore Trial. Blood 136:34-35. <https://doi.org/10.1182/blood-2020-134912>

10. Cortes JE, Kantarjian HM, Mauro MJ et al (2021) Long-term cardiac, vascular, hypertension, and effusion safety of bosutinib in patients with Philadelphia chromosome-positive leukemia resistant or intolerant to prior therapy. Eur J Haematol 106:808-820. <https://doi.org/10.1111/ejh.13608>

11. Cortes JE, Milojkovic D, Gambacorti-Passerini C et al (2022) Bosutinib (BOS) in newly diagnosed chronic myeloid leukemia (CML): gastrointestinal (GI), liver, effusion, and renal safety characterization in the BFORE trial. JCO 40(Suppl 16):7049. <https://doi.org/10.1200/JCO.2022.40.16_suppl.7049>

12. Cortes JE, Gambacorti-Passerini C, Kim D-W et al (2017) Effects of Bosutinib Treatment on Renal Function in Patients With Philadelphia Chromosome-Positive Leukemias. Clin Lymphoma Myeloma Leuk 17:684-695.e686. <https://doi.org/10.1016/j.clml.2017.06.001>

13. Claudiani S, Janssen JJWM, Byrne J et al (2022) A retrospective observational research study to describe the real-world use of bosutinib in patients with chronic myeloid leukemia in the United Kingdom and the Netherlands. Eur J Haematol 109:90-99. <https://doi.org/10.1111/ejh.13775>

14. García-Gutiérrez V, Milojkovic D, Hernandez-Boluda JC et al (2019) Safety and efficacy of bosutinib in fourth-line therapy of chronic myeloid leukemia patients. Ann Hematol 98:321-330. <https://doi.org/10.1007/s00277-018-3507-2>

15. Apperley JF, Byrne JL, Smith G et al (2016) The real world use of bosutinib in patients with chronic myeloid leukaemia. Blood 128:5435. <https://doi.org/10.1182/blood.V128.22.5435.5435>
